# Lipopolysaccharides Drive Proinflammatory Extracellular Vesicle Secretion in Coronary Artery Endothelial Cells via Noncanonical Inflammasome Activation

**DOI:** 10.1007/s00018-025-06006-y

**Published:** 2026-04-21

**Authors:** Katariina Nurmi, Martina B. Lorey, Jukka Parantainen, Wojciech Cypryk, Eirini Kalogerakou, Vesa-Petteri Kouri, Juha Kaivola, Marcelina Bilicka, Arzu Beklen, Yan Chen, Maria Stensland, Sampsa Matikainen, Tuula A. Nyman, Kari K. Eklund

**Affiliations:** 1 Faculty of Medicine, Translational Immunology Program, University of Helsinki, Clinicum, Helsinki, Finland; 2 Atherosclerosis Research Laboratory, Wihuri Research Institute, Helsinki, Finland; 3 Faculty of Biological and Environmental Sciences, Molecular and Integrative Biosciences Research Programme, University of Helsinki, Helsinki, Finland; 4 Centre of Molecular and Macromolecular Studies, Polish Academy of Sciences, Lodz, Poland; 5 Faculty of Dentistry, Eskisehir Osmangazi University, Eskisehir, Turkey; 6 Urological department, Institute of Clinical Medicine, the First Affiliated Hospital of Zhengzhou University, Zhengzhou City, Hennan Province China; 7 Department of Immunology, University of Oslo and Oslo University Hospital, Oslo, Norway; 8 Department of Rheumatology, University of Helsinki and Helsinki University Hospital, Helsinki, Finland; 9 ORTON Orthopaedic Hospital, Helsinki, Finland

**Keywords:** Coronary artery disease, Lipopolysaccharide, Caspase-4/-5, Dysbiosis, Bacterial outer membrane vesicle, Arterial endothelium, MLKL

## Abstract

Intestinal dysbiosis and impaired gut barrier function lead to increased blood lipopolysaccharide (LPS) load, a condition known as endotoxemia. We investigated whether intracellular LPS induces activation of the noncanonical caspase-4 and -5 inflammasome in human coronary artery endothelial cells (HCAECs) and whether this contributes to arterial wall inflammation. Cytosolic LPS induced activation of the noncanonical inflammasome in endothelial cells, leading to marked increase in extracellular vesicle (EV) secretion. These EVs were enriched with biologically active proteins, particularly kinases, involved in bacterial invasion, cell survival, and pyroptosis. EV secretion was dependent on the pore forming proteins Gasdermin D and Mixed lineage kinase domain-like pseudokinase (MLKL). Intracellular LPS also upregulated the expression of adhesion molecules, which increased monocyte attachment to HCAECs. EVs from activated endothelial cells stimulated expression of proinflammatory cytokines and interferon-stimulated genes in macrophages. Ultimately, noncanonical inflammasome activation increased endothelial cell pyroptosis. These findings suggest that activation of the noncanonical inflammasome in endothelial cells could represent a novel link between intestinal dysbiosis and the ensuing endotoxemia and arterial wall inflammation.

**Graphical Abstract:**

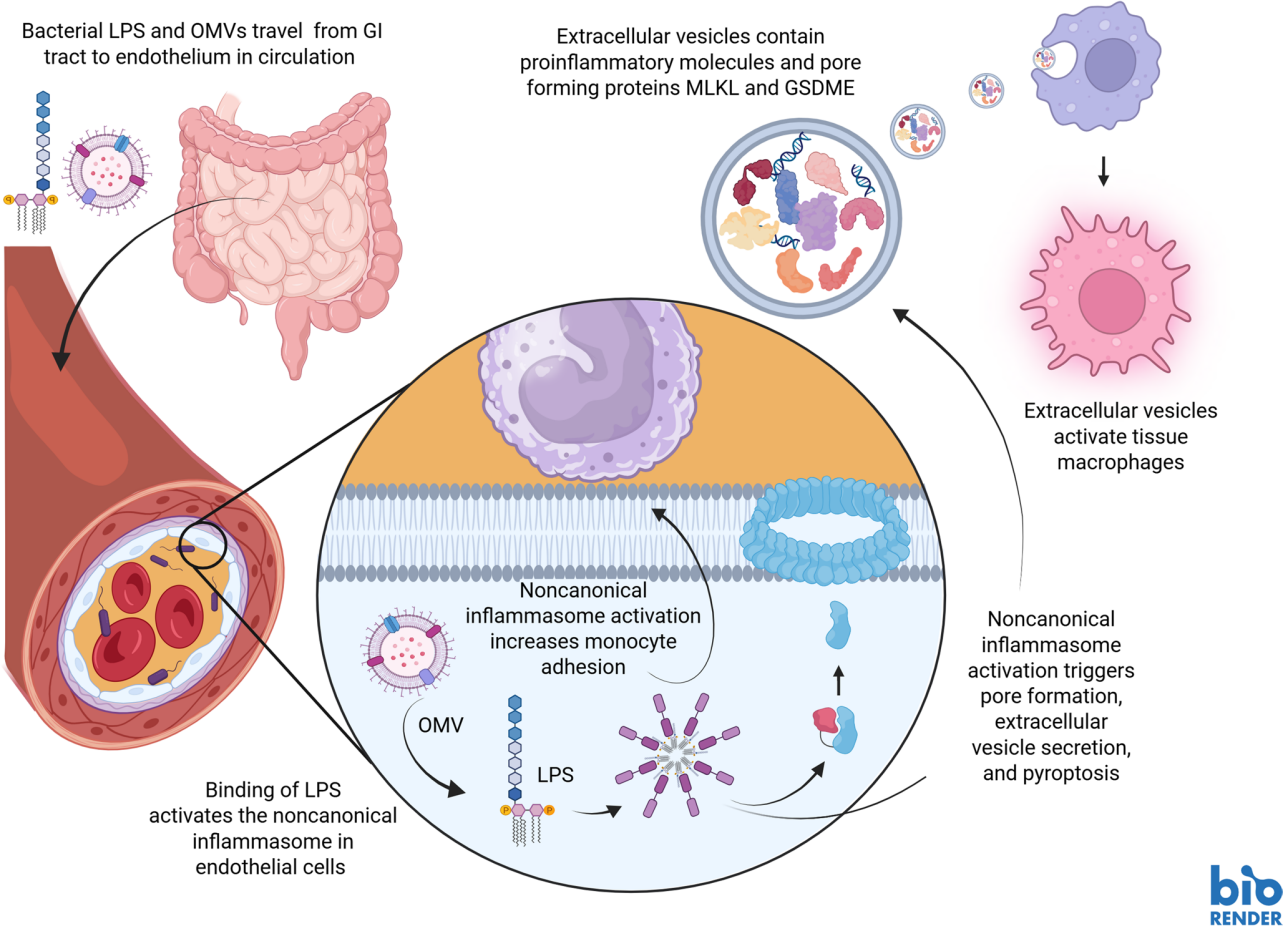

**Supplementary Information:**

The online version contains supplementary material available at 10.1007/s00018-025-06006-y.

## Introduction

Imbalance in the composition of intestinal microbiota (dysbiosis) is associated with intestinal inflammation and impaired gut barrier function, which increase blood levels of bacterial metabolites, such as trimethylamine-N-oxide, and bacterial structural components, in particular lipopolysaccharides (LPS). Increased level of circulating LPS is referred to as endotoxemia, which imposes a constant low grade proinflammatory burden on the body. Both increased gut permeability and intestinal dysbiosis have been associated with the progression of atherosclerotic cardiovascular diseases (ASCVD) in animal models and in patients [1–3]. The causal relations between ASCVD and gut microbiota and molecular mechanisms that mediate disease progression are however, yet to be elucidated.

Most human cells are equipped with a wide variety of patten recognition receptors that recognize diverse pathogen- and host-derived danger-associated molecules and sense changes in cell homeostasis. Toll-like receptors (TLR) 4 and 2 bind extracellular LPS, which consequently trigger transcription of proinflammatory cytokines and interferons. In addition to cell surface receptors, also intracellular receptors sense LPS. LPS can access the cytosol by several mechanisms. Under stressed conditions, such as in contact with host tissues or during dysbiosis, bacteria increase secretion of LPS-containing outer membrane vesicles (OMV) which are taken up by the cells [4]. LPS can bind to extracellular vesicles (EV) [5] or to endogenous molecules such as high mobility group box 1 (HMGB1) protein [6], both of which can deliver LPS into the cells and are abundant in circulation. Once in the cytosol, LPS activates a special class of pattern recognition receptors called inflammasomes. They are large cytosolic protein complexes that promote inflammation in response to endogenous danger-associated molecular patterns or exogenous pathogen-associated molecular patterns [7]. Cytosolic LPS directly binds to caspase-4/5, which nucleates the noncanonical inflammasome in human. Noncanonical inflammasome assembly leads to activating autocleavage of the procaspases 4 and 5, which consequently cleave gasdermin D (GSDMD). The cleaved N-terminal fragment of GSDMD forms pores on the plasma membrane causing the secretion of various cytosolic molecules and proinflammatory mediators, including the bulk secretion of inflammasome-dependent cytokines interleukin (IL)-1β and IL-18, and initiates lytic cell death termed pyroptosis [8, 9]. K^+^ efflux through GSDMD pores promotes the assembly and activation of the nucleotide-binding oligomerization domain, leucine rich repeat-containing protein 3 (NLRP3) inflammasome [10]. The NLRP3 inflammasome sensor protein, NLRP3, can be activated by a wide range of stimuli, including pathogen- and danger-associated molecules, as well as disruptions in cellular homeostasis [10]. Perturbations in ion homeostasis are thought to represent the common pathway through which most, if not all, NLRP3 activators converge [11]. Active NLRP3 inflammasome cleaves procaspase-1, which then cleaves GSDMD and the proinflammatory cytokines proIL-1β and proIL-18 into their active forms. In contrast, the noncanonical inflammasome caspases, caspase-4 and -5, cleave GSDMD and IL-18 into their biologically active forms, but do not generate mature IL-1β [12]. 

High levels of LPS observed during septic shock have been shown to induce pyroptosis and NLRP3 inflammasome activation in lung endothelial cells leading to progression of an acute lung injury in vivo in mice [6, 13, 14]. Cheng et al. showed that transfected LPS induces pyroptosis, and secretion of IL-1β from mouse and human microvascular endothelial cells [14]. In mice, LPS transfection and endotoxemia also induced caspase-11-dependent cleavage of GSDMD and EV secretion [14]. 

Strategically positioned at the interface of blood and tissues, endothelial cells contribute to body´s immune defense not only as a barrier, but also as regulators of immune responses [15, 16]. The endothelial lining is the first layer which circulating cells and molecules encounter on their way into the tissue. Bacterial DNA and other bacterial components, including LPS, have been found in atherosclerotic plaques. This indicates the ability of components of the oral and gut microbiota to cross the endothelial barrier and to migrate from blood to surrounding tissue [17]. These transmigrating molecules can activate endothelial cells, thus modulating the recruitment of leukocytes, interactions between endothelial cells, and the crosstalk between endothelial cells and the adjacent tissues. Endothelial cells secrete a variety of molecules that mediate communication with other cells, and many of these endogenous mediators, including damage-associated molecular patterns and extracellular vesicles (EV), have been implicated in the pathogenesis of ASCVD. EVs are heterogenous population of lipid bilayer vesicles, consisting of exosomes (typically 50–100 nm), ectosomes (typically 100–350 nm), and apoptotic bodies (typically 500 nm–2 μm) [18]. EVs are secreted practically by all cell types, in both resting and activated state. EVs carry a wide variety of metabolites, lipids, proteins, and nucleic acids, and the activation state of the cell has a significant impact on the EV content [19]. The outer membrane of EVs is derived from the donor cell membrane and includes the membrane molecules, which can activate receptors on the recipient cell´s membrane. The internal cargo can be directly delivered to the recipient cell´s cytoplasm through EV fusion with the cell membrane or via endocytosis, where the cargo proteins may be degraded [20]. The endothelium has a particularly rich vesicular network allowing endothelial cells to produce the second biggest volume of circulating EVs after platelets [19]. 

Here, we show that in human coronary endothelial cells (HCAEC) and human umbilical vein endothelial cells (HUVEC) activation of noncanonical inflammasome by transfecting liposome encapsulated LPS or by exposing the cells to *E. coli* OMVs induces expression of vascular cell adhesion molecule (*VCAM1*), leading to increased adhesion of monocytes, and robust pore forming protein -dependent secretion of proteins in the EV fraction followed by caspase-4/5 mediated cell death. We further show that the endothelial cell-derived EVs promote activation of macrophages and induce expression of proinflammatory cytokines and interferon response in human primary macrophages thus potentially promoting vascular inflammation.

## Materials and methods

### Cell culture

Human coronary artery cells (HCAEC) were purchased from PromoCell (cat. C-12221). HCAECs were cultured in Endothelial Cell Basal Medium MV2 (PromoCell, C-22121), supplemented with 100 U/mL penicillin, 100 µg/mL streptomycin (PS, Gibco, cat.15140-122), and the included supplement pack according to manufacturer’s recommendations. Human umbilical vein endothelial cells (HUVEC, ATCC, cat. CRL1730) were cultured in RPMI 1640 (EuroClone, cat. ECB9006L), supplemented with 10% fetal bovine serum (FBS; Gibco, cat. 10500-064), PS, and 2 mM l-glutamine (l-glut, Gibco, cat. 35050-038).

THP-1 monocytes (ATCC, cat. TIB-202) were cultured in RPMI supplemented with 10% FBS, PS, l-glut, and 25 mM HEPES (Fischer bioreagents, BP299-500). Human primary macrophages were differentiated from blood mononuclear cells isolated from Finnish Red Cross Blood Service (Helsinki, Finland) peripheral blood buffy coats. Peripheral blood was donated by healthy volunteers, who had signed an informed consent. Buffy coats were by-products of blood preparations, and their allocation to scientific purposes was approved by Finnish Red Cross Blood Service (permit no. 3/2023). Blood mononuclear cells were extracted by density gradient centrifugation using Ficoll-Paque PLUS (GE Health care, cat. 17-1440-03). The obtained mononuclear cell layer was collected, and the cells were washed four times with PBS-/-. Mononuclear cells were counted with a TC20 automated cell counter (Bio-Rad, Hercules, CA) and 1.5 × 10^6^ cells were plated per well on 24-wells or 6 × 10^6^ cells per well on 6-well plates in DMEM supplemented with PS. Monocytes were allowed to attach for 60 min and the non-adherent cells were removed by washing twice with PBS-/-. Monocytes were differentiated to macrophages by culturing them for 7 days in the presence of recombinant human granulocyte-macrophage colony-stimulating factor (GM-CSF, 10 ng/mL, Miltenyi Biotec, cat. 130-093-865) in macrophage serum free medium (SFM, Gibco, cat. 12065-074) supplemented with PS. During differentiation human monocyte-derived macrophages were washed with PBS-/- and SFM was changed; first wash 24 h after plating and then after every 48 h. Differentiated macrophages were used for experiments after 6–7 days of culture, the last washes and media change was performed 20 h before the experiment.

All cell types were maintained in humidified incubator at 37 °C and 5% CO_2_.

### Cell activations and gene silencing

Before experiments, HCAECs were detached using Detach Kit (Promocell, C-41210) according to manufacturer’s recommendations, and seeded on 0.015 × 10^6^ cells/well on 96-wells, 0.075 × 10^6^ cells/well on 24-wells or 0.3 × 10^6^ cells/well on 6-wells. The cells were allowed to grow 48–72 h (until confluent) in MV2 media. Before the experiment the cells were washed twice with PBS -/- and changed to Opti-MEM media (Gibco, cat. 31985-047), supplemented with only PS, except for the data in Fig. S1 and S4 where the effect of serum on expression of NLRP3 and pro-IL-1β was assessed.

HUVECs were detached by washing the cells two times with PBS -/-, after which 2.5% trypsin (Gibco, cat. 15090-046) was added and the detachment was monitored under a microscope. Trypsin was inactivated by adding culture media containing the 10% FBS. 0.3 × 10^6^ cells/well were plated on 6-well plates and grown until confluent. On the day of experiment the cells were washed three times with PBS -/- and supplemented with fresh RPMI media supplemented with PS and l-glut.

For NLRP3 activations THP-1 monocytes were washed with PBS-/- and placed in RPMI supplemented with PS, l-glut, and HEPES. THP-1 monocytes were primed with *E. coli* O111:B4 LPS 1 µg/mL (Sigma, cat. L3012) for 5 h, after which 4 µM nigericin (Sigma Aldrich, cat. N7143) was added for 1 h. One day before stimulation, human monocyte-derived macrophages were changed into new GM-SFM media and left to rest for 20 h.

For LPS time point experiments, the cells were activated with extracellular O111:B4 LPS (Sigma, cat. L3012) for 1.5 h, 3 h or 6 h. To activate the caspase-4/5 inflammasome, ultra-pure smooth O111:B4 LPS 2 µg/ml (Invivogen, cat. tlrl-3pelps) was transfected into cells using 5 µl/ml Lipofectamine 2000 (Invitrogen, cat. 11668019), Lipofectamine 2000 alone was used as mock treatment. Also *E. coli* OMVs (Invivogen, cat. tlrl-omv-1) were used for noncanonical inflammasome activations. HCAECs were stimulated for 16 h in the presence of OMVs (10 µg/mL) in Opti-MEM. Inhibitors of caspase-4 Ac-LEVD-CHO (ICH-2, 25 µg/ml, Sigma Aldrich cat. SCP0087) and caspase-1 and 4, Z-YVAD-FMK (5 µM, R&D Systems, cat. FMK005), and 10, 30 or 50 µM inhibitor of MLKL, Necrosulfonamide (Merck, cat. 480073), or small molecule NLRP3 inhibitor, Cy-09, (10 µM, Glixx Laboratories Inc, cat. GLXC-10947), were added to the cells 1 h before transfection.

For siRNA transfections, HCAECs and HUVECs were cultured to 80–90% confluency, washed twice with PBS-/- and placed in Opti-MEM. A complex of RNAiMAX (6 µl/mL, Invitrogen, cat. 13778-075) and siRNA (60 pmol/mL) for MLKL (Dharmacon on-target plus smart pool, cat. L-005326-00-0005), CASP4 (Dharmacon on-target plus smart pool, cat. L-004404-00-0005), GSDMD (Dharmacon on-target plus smart pool, cat. L-016207-00-0005) or non-targeting siRNA for mock transfections (Dharmacon on-target plus control pool, cat. D-001810-10-05) was added dropwise on the cells. 6 h after transfection complexes were removed and the cells were incubated in their culture media for 48 h (HUVECs) or 72 h (HCAECs) before activations.

### Endothelial cell extracellular vesicle purification and macrophage stimulations

HCAECs were cultured in T75 bottles until 80–90% confluency in Endothelial Cell Basal Medium MV2, supplemented with PS. Seven T75 bottles were transfected with LPS and Lipofectamine 2000 for 6 h, and seven bottles received Lipofectamine 2000 alone for 6 h as a mock treatment. After activation detached cells and cell remnants were removed by centrifugation at 500 g for 10 min at 4 °C, subsequently media were transferred to a new falcon tube and centrifugated at 3000 g, for 30 min at 4 °C. EVs were collected by ultracentrifugation of cell supernatant at 100 000 g for 2 h at 4 °C followed by washing of the EVs by resuspension in PBS and ultracentrifugation at 100 000 g for 2 h at 4 °C, using SW40 rotor and Beckman Optima LE-80 K centrifuge. The EV pellets were resuspended in 210 µl of PBS and stored at 4 °C. EV preparations from LPS and mock transfected HCAECs were used for nanoparticle tracking analysis (ZetaVIEW S/N 20–572, software ZetaView, version 8.05.12 SP2) and 60 µl/ml of EV preparations were used for macrophage activations. As a control for possible remnants of biologically active LPS, macrophages were stimulated extracellularly with 0.87 ng of ultra-pure LPS, which was detected in the EV preparations of LPS-transfected HCAECs (0.66–0.87 ng/mL) by EndoLISA (bioMérieux, cat. 609033), also 100 times (87 ng/mL) and 10 000 times (8700 ng/mL) larger LPS concentrations were used. To control for possibly remaining remnants of transfection complexes in the EV preparations, macrophages were stimulated with freshly prepared LPS-lipofectamine transfection complexes or lipofectamine alone as a mock treatment for 6 h.

### Global secretome analysis

Label-free quantitative proteomics was used to identify and quantify proteins secreted in response to LPS transfection. Five biological replicates were included in the analysis. Total secretomes were fractionated into two fractions using size-exclusion filtration as previously described [21]. First, equal volumes of cell culture media were concentrated using 100 kDa Amicon centrifugal concentrators (Merck, cat. UFC910024) to obtain the fraction containing extracellular vesicles (‘EV fraction’). Subsequently, equal volumes of flow-through from 100 kDa size exclusion filtration were concentrated with 10 kDa Amicon centrifugal concentrators (Merck, cat. UFC901024); this fraction contains proteins secreted in a soluble form (rest secretome, ‘RS fraction’). Proteins in the EV fraction were precipitated with ice cold acetone with 1 M HCl in −20 °C o/n and dissolved in 6 M urea/100 mM ammonium bicarbonate. After that proteins from both EV and RS fractions were reduced, alkylated and digested with trypsin (Promega). The resulting peptides were transferred to a new tube, acidified, and desalted by the STAGE-TIP method using a 3 M Empore™ C18 resin disc.

LC-MS/MS analysis was carried out with nEASY-LC coupled to QExactivePlus (Thermo) with EASY Spray PepMap^®^RSLC C18-column using a 60 min separation gradient. Raw files from LC-MS/MS analyses were submitted to MaxQuant software (version 1.6.1.0) for protein identification and label-free quantification. Parameters were set as follows: Carbamidomethyl (C) was set as a fixed modification and protein N-acetylation and methionine oxidation as variable modifications. First search error window was 20 ppm and main search error 4.5 ppm. Trypsin without proline restriction enzyme option was used, with two allowed miscleavages. Minimal unique peptides were set to one, and the allowed FDR was 0.01 (1%) for peptide and protein identification. The Uniprot human database was used. Generation of reversed sequences was selected to assign FDR rate. Known contaminants and reversed entries as provided by MaxQuant and identified in samples were excluded from further analysis. For additional data filtering and statistical analysis MaxQuant proteinGroup-file was further analyzed in Perseus [22] software (version 1.6.15.0). In Perseus, the intensity values were log10 transformed, data was filtered to include only reproducible identifications (minimum 3/5 identifications in at least one experimental group), missing values were imputed with constant = 0, and for statistical analysis paired t-test with permutation-based FDR < 0.05 was used.

The proteomic datasets were submitted to Ingenuity Pathway Analysis software (IPA, Ingenuity Systems, Mountain View, CA, www.ingenuity.com). Analysis settings were Student´s T-test cutoff p-value 0.05, expr false discovery rate (q-value) 0.05; expr log ratio down − 1 and up 3. For selection of top affected canonical pathways, they were sorted by –log (p-value) showing only –log(p-values) greater than 1.3 and they were visualized partly with SRplot [23]. In addition, the interactomes within the proteomic datasets were analyzed with STRING [24]. 

### Analysis of cytokine secretion and lactate dehydrogenase release

Upon sample collection cell culture media were centrifuged 300 g 5 min, aliquoted and stored at −80 C for further use. Elisa for human Total IL-18 DuoSet ELISA (R&D Systems, cat. DY318-05) and mature human IL-1β/IL-1F2 (R&D Systems, cat. DY201) were used for detection of secreted IL-18 and IL-1β from cell culture supernatants. The release of lactate dehydrogenase (LDH) was measured from cell culture supernatants using the Cytotoxicity Detection Kit (LDH) (Roche, Diagnostics, cat. 11644793001). The assays were performed according to manufacturer’s protocols.

### Quantitative real-time RT-PCR

RNA was isolated using RNeasy Plus Mini Kit (Qiagen, cat. 74136), and synthetized to cDNA with iScript kit (Bio-Rad, cat. 170–8891). Quantitative real time reverse transcriptase PCR (qPCR) was performed from 5 ng of cDNA per reaction using 5X HOT FIREPol EvaGreen qPCR SuperMix (SolisBiodyne, cat. 08–25-00001–10) and LightCycler96 instrument (Roche). Primer sequences are presented in Supplementary Table 1. The target gene expression was calculated relative to the housekeeping gene RPLP0 using the 2^(-ΔΔCt) method and is presented as arbitrary units (a.u.), representing the fold change in expression induced by the treatment compared to the untreated control.

### SDS-page and Western blotting

HCAECs and HUVECs were scraped into ice-cold 1 x cell lysis buffer (Cell Signaling Technology, cat. 9803S) supplemented with 1x protease inhibitor cocktail (Roche, cat. 04 693 116 001) and 1x phosphatase inhibitor cocktail (Pierce, cat. A32957). The lysates were homogenized by bath sonication for 3 × 15 s on ice and mixed with SDS-PAGE Laemmli loading buffer (Bio-Rad) reduced with 1.25 M DTT, and the proteins were denatured by cooking at 95 °C, 5 min. SDS-PAGE was run with gels prepared with TGX Stain-Free FastCast Acrylamide Kit 7.5% and 10% (Bio-Rad, cat. 1610181 and 1610183), and the loading was controlled by stain-free protein imaging. The proteins were transferred onto PVDF membranes using Transblot Turbo Transfer System and reagents according to manufacturer’s recommendations (Bio-Rad). PVDF membranes were blocked with either non-fat milk or BSA in TBST 0.05%, depending on the recommendations of the primary antibody suppliers, and incubated overnight at + 4 °C with primary antibodies against NLRP3 (AdipoGen, cat. AG-20B-0014), MLKL (Abcam EPR17514, cat. ab184718), proIL-1β (Santa Cruz Biotechnology, cat. sc-7884), and N-terminal GSDMD (Cell Signaling, cat. 36425S). Bands were detected by using HRP-conjugated secondary antibodies (anti-rabbit ab, cat. P0448 or anti-mouse cat. P0447, both from Dako), after 1 h incubation at RT, followed by detection using Clarity Western ECL Substrate (Bio-Rad, cat. 170–5061) and ChemiDoc MP Imaging System (Bio-Rad).

Total secreted proteins were concentrated from equal volumes of cell supernatant with 100 kDa or 10 kDa Amicon centrifugal concentrators, equal volumes of concentrated media were separated by SDS-PAGE, and proteins were visualized with silver-staining [25]. 

### Monocyte adhesion

The monocyte experiment was performed as previously described [26], except that THP-1 monocytes were used instead of human primary macrophages and the experiments were performed on 0.1% gelatin (Merck, cat. ES-006-B) coated black 96-well ViewPlates (Revvity, cat. 6005182) to allow microscopy. Briefly: HCAECs were plated 15 000 cells/well in MV2 media and let to grow to 80–90% confluency (usually for 24 h). Before stimulations the cells were washed twice with PBS -/- and changed in Opti-MEM media, supplemented with PS. Ultra-pure *E. coli* O111:B4 LPS (2 µg/ml) was transfected into cells using Lipofectamine 2000 (5 µl/ml) for 2 h or HCAECs were activated with *E. coli* OMVs (10 µg/ml, 2 h), Lipofectamine or vehicle alone was used as mock treatment. Vascular cellular adhesion molecule-1 (VCAM1) (InVivoMAb anti-mouse CD106, cat. BE0027) neutralizing antibody or its IgG-control (InVivoMAb rat IgG1, cat. BE0088) 5 µg/mL were applied on the cells 2 h before activation with OMVs. THPs were stained with 2 µM eBioscience™ Calcein AM fluorescent dye, ultra-pure grade (Invitrogen, cat. 65–0853-78) for 30 min at 37 °C in RPMI w/o serum, after which THP-1 monocytes were washed twice with PBS to remove excess staining and resuspended in Opti-MEM. 2 h after stimulation HCAECs were washed twice with PBS and 0,25 M THP-1 monocytes in 100 µl/well were added on HCAEC monolayer and let adhere for 1 h. To exclude the effect of possible remaining transfection particles or OMVs equal dose of LPS-lipofectamine complexes or OMVs, which were used to stimulate HCAECs, were added to the culture when THP monocytes were combined with HCAECs. After 1 h coculture nonadherent THP-1 monocytes were removed by washings with PBS x 3, and the fluorescence was detected by microscopy. Fluorescent cells were counted from 4 (OMV) or 8 (LPS transfection) biological replicates, each with 5 technical replicate wells (2 images were taken per technical replicate). Zoe Fluorescent Cell Imager (Bio-Rad) was used for imaging, and the numbers of adherent fluorescent cells were analyzed with FIJI ImageJ 2.1.0/1.54f/Java 1.8.0_172 (64-bit) for Lipofectamine transfections. Image analysis of OMV activations was performed with CellProfiler 4.2.8 [27]. The whole analysis pipeline is detailed in Supplementary File 1. In brief, illumination correction was calculated with “Fit polynomial” method and applied on the images. Primary objects were identified with Otsu global thresholding with three classes. Identified objects were then filtered based on roundness (formfactor > 0.5) and machine vision as well as illumination features were exported to a.csv file.

### Statistical analyses

Statistical comparison between the experimental groups was conducted using GraphPad Prism version 9 (GraphPad Software, La Jolla, CA), the used statistical analysis and post hoc tests are implicated in the figure legends. The data is shown as mean ± SD, statistical significance was set at *P* < 0.05 (*P < 0.05, ** *P* < 0.01, ***P < 0.001, ****P < 0.0001). With proteomics data paired t-test with permutation-based FDR < 0.05 as the criteria for statistical significance was used. All experiments with cell lines were done with two technical replicates, except for the blots (no technical replicates) and immunofluorescence stainings (5 technical replicates).

## Results

### Intracellular LPS induces cell death and secretion of IL-18 in human endothelial cells

**Fig. 1:**
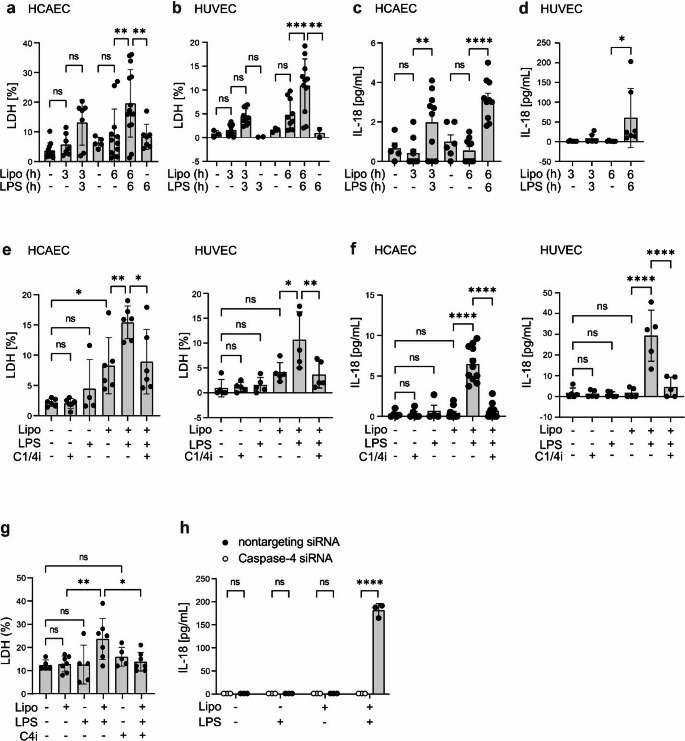
**Intracellular LPS induces noncanonical inflammasome activation and subsequent pyroptotic cell death in endothelial cells. **HCAECs and HUVECs were transfected with a complex of ultra-pure LPS (2 µg/ml) and lipofectamine (5 µl/ml) or mock-treated with lipofectamine (5 µl/ml) for indicated times and secretion of a-b) LDH was detected by LDH assay, and c-d) IL-18 was detected by ELISA. HCAECs and HUVECs were treated with caspase-1/4 inhibitor Z-YVAD-FMK (5 µM) for 1 h prior to transfection with a complex of ultra-pure LPS (2 µg/ml) and lipofectamine (5 µl/ml) or mock-treated with lipofectamine (5 µl/ml) for 6 h and secretion of e) LDH and f) IL-18 were measured. g) HCAECs were treated with caspase-4 inhibitor ICH-2 (25 µM) for 1 h prior to transfection with a complex of ultra-pure LPS (2 µg/ml) and lipofectamine (5 µl/ml) or mock-treated with lipofectamine (5 µl/ml) for 6 h. Secretion of LDH was detected. h) HUVECs were transfected with caspase-4 siRNA or mock siRNA (30 pmol/ml) using RNAiMAX (3 µl/ml), and 48 h later noncanonical inflammasome was stimulated by transfection of ultra-pure LPS (LPS 2 µg/ml and/or lipofectamine 5 µl/ml, 6 h). Secretion of IL-18 was detected. Statistics: One-way ANOVA followed by Sidak´s multiple comparisons test. a-b) HCAEC: *n* = 5–14, HUVEC *n* = 2–14. c-d) HCAEC: *n* = 5–10, HUVEC *n* = 6–7. e) HCAEC: *n* = 4–6, HUVEC *n* = 5. f) HCAEC: *n* = 5–11, HUVEC *n* = 5. g) *n* = 5–7, h) *n* = 3

In humans, noncanonical inflammasome activation is induced by direct binding of LPS lipid A moiety to caspase-4/5 [9, 28]. The hallmarks of inflammasome activation are pyroptotic cell death and secretion of alarmins and mature forms of proinflammatory cytokines IL-1β and IL-18 [29]. We first studied whether noncanonical inflammasome activation induces pyroptotic cell death in human primary endothelial cells. Transfection of HCAECs with ultra-pure LPS increased cell death to some extent already at the 3 h time point as measured by release of LDH (Fig. 1a). LDH release further increased towards the 6 h time point, at which time point the cell death was statistically significantly increased. Increase of cell death was equally evident in human umbilical cord endothelial cells (HUVEC) at 3 h and 6 h after LPS transfection (Fig. 1b). Transfected LPS also induced IL-18 secretion both in HCAECs and HUVECs, although the amounts of IL-18 secreted by HCAECs were low (Fig. 1c, d).

Next, we studied the effect of caspase-1/4 inhibitor (Z-YVAD-FMK) on cell death and secretion of IL-18. Z-YVAD-FMK significantly reduced cell death and IL-18 secretion both in HCAECs and HUVECs (Fig. 1e, f). Lipofectamine treatment alone increased cell death borderline significantly in Fig. 1e. However, pooling the 6 h LDH data from experiments shown in panels 1a, e, and g, to get larger number of biological replicates, showed that lipofectamine treatment does not induce cell death (Fig. S5a). Also, the specific caspase-4/5 inhibitor Ac-LEVD-CHO inhibited cell death in HCAECs (Fig. 1g), and in HUVECs silencing of caspase-4 blocked the secretion of IL-18 (Fig. 1h). Taken together, these results show that pyroptosis and IL-18 secretion are dependent on caspase-4/5 activation in human endothelial cells.

### Human primary coronary artery endothelial cells express noncanonical inflammasome components but not the components of the NLRP3 inflammasome

**Fig. 2:**
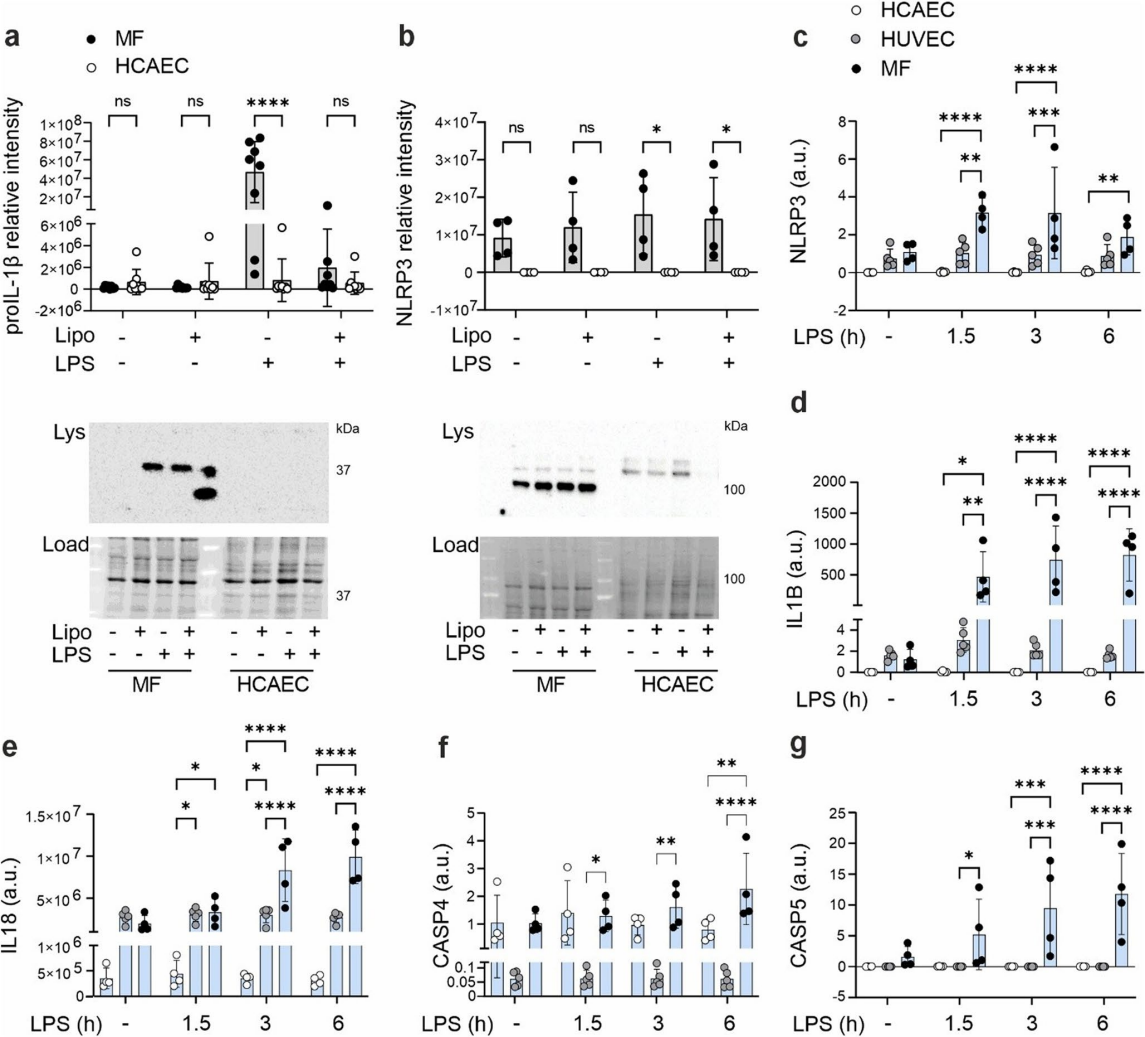
**Primary human endothelial cells express noncanonical inflammasome proteins but not IL-1β or NLRP3 inflammasome proteins**. HCAECs and human primary macrophages were transfected with a complex of ultra-pure LPS (2 µg/ml) and lipofectamine (5 µl/ml) or mock-treated with lipofectamine (5 µl/ml) or stimulated with extracellular LPS (1 µg/ml) for 6 h and protein expression of **(a)** IL-1β and **(b)** NLRP3 was analyzed by Western blotting from cell lysates. Representative Western blots and loading are shown. HCAECs, HUVECs and human primary macrophages were stimulated with extracellular LPS for indicated times and mRNA expression of inflammasome pathway proteins **(c)***NLRP3*, **(d)***IL1B*, **(e)***IL18*, **(f)***CASP4*, and **(g)***CASP5* was analyzed by RT-qPCR. Statistics: Two-way ANOVA followed by Sidak´s multiple comparisons test. **a**) *n* = 8, **b-g**) *n* = 4

Following the non-canonical inflammasome activation, we did not observe secretion of IL-1β (Fig. S1b), but we observed consistent secretion of IL-18 from HCAECs, although the secretion was low compared to that observed in HUVECs, and to the levels previously reported for human macrophages [21]. Since activation of the NLRP3 inflammasome is required for the bulk secretion of inflammasome-dependent cytokines, we next analyzed the expression of NLRP3 inflammasome components in endothelial cells and compared their expression to that in macrophages, using the same stimulation times and LPS concentrations. In humans, however, NLRP3 inflammasome activation is not mandatory for the secretion of IL-18, as also caspase-4 processes proIL-18 into its secreted form [12]. In serum-free media no protein expression of proIL-1β or NLRP3 was observed in HCAECs with or without LPS stimulation, while in macrophages proIL-1β expression was induced by LPS (Fig. 2a, b). NLRP3 was already expressed on baseline in untreated macrophages, and it was further increased by LPS stimulation. Also on RNA level, stimulation with extracellular LPS induced increasing expression of *NLRP3* and *IL1B* in macrophages starting from the 1.5 h time point, but no expression of *NLRP3* or *IL1B* was detected in HCAECs (Fig. 2c, d). HUVECs differed notably from HCAECs, as they expressed *NLRP3* and *IL1B*, although at significantly lower levels than primary macrophages. In HCAECs and HUVECs, expression of *IL18* was detected but at lower level compared to macrophages, and unlike in macrophages, the expression was not increased with LPS stimulation. (Fig. 2e). The noncanonical inflammasome receptor protein *CASP4* was expressed on a similar level both in HCAECs and macrophages, and somewhat surprisingly, at lower level in HUVECs (Fig. 2f). However, for a constitutively expressed gene, *CASP4* expression in HUVECs was still relatively high (average Ct ~ 24, while in HCAECs Ct was ~ 20 and in macrophages Ct ~ 21). *CASP5* was not expressed in HCAECs or HUVECs, and its expression could not be induced by extracellular LPS (Fig. 2g). Taken together, unlike in human primary macrophages, neither intra- nor extracellular LPS induced NLRP3 or IL-1β expression in primary endothelial cells, HCAECs. In contrast, the human endothelial cell line (HUVECs) expressed NLRP3 inflammasome proteins, although at lower levels compared to macrophages.

To further study the expression of NLRP3 inflammasome components in HCAECs we stimulated the cells in the presence of serum. *NLRP3* and *IL1B* expression is induced by activation of NF-κB signaling following TLR or cytokine receptor activation in a process called “priming” [30]. Human endothelial cells cannot be primed with extracellular LPS in serum free media as they do not express an integral part of the LPS receptor complex, the LPS-binding cluster of differentiation 14, on their cell membrane [31]. In contrast, TLR4 signaling can be induced in the presence of serum, which provides soluble cluster of differentiation 14 and lipopolysaccharide binding protein [31]. However, neither extracellular or intracellular LPS with or without serum could induce protein or gene expression of *IL1B* or *NLRP3* in HCAECs (Fig. S1c, d). To confirm that TLR4 was activated in serum containing media, we analyzed the expression of interleukin-6 (*IL6*) and chemokine (C-C motif) ligand 2 (*CCL2)*, which have been previously shown to be induced by extracellular LPS in HCAECs [32]. Indeed, extracellular LPS increased *IL6* and *CCL2* expressions, and even though expression of *CCL2* was modestly induced also in serum free media, in the presence of serum expressions of both *IL6* and *CCL2* were significantly increased (Fig. S1e). Expression of *CASP4* was not affected by serum (Fig. S1f).

### Noncanonical inflammasome activation with intracellular LPS or *E. coli* outer membrane vesicles induces robust EV-mediated protein secretion from endothelial cells

**Fig. 3:**
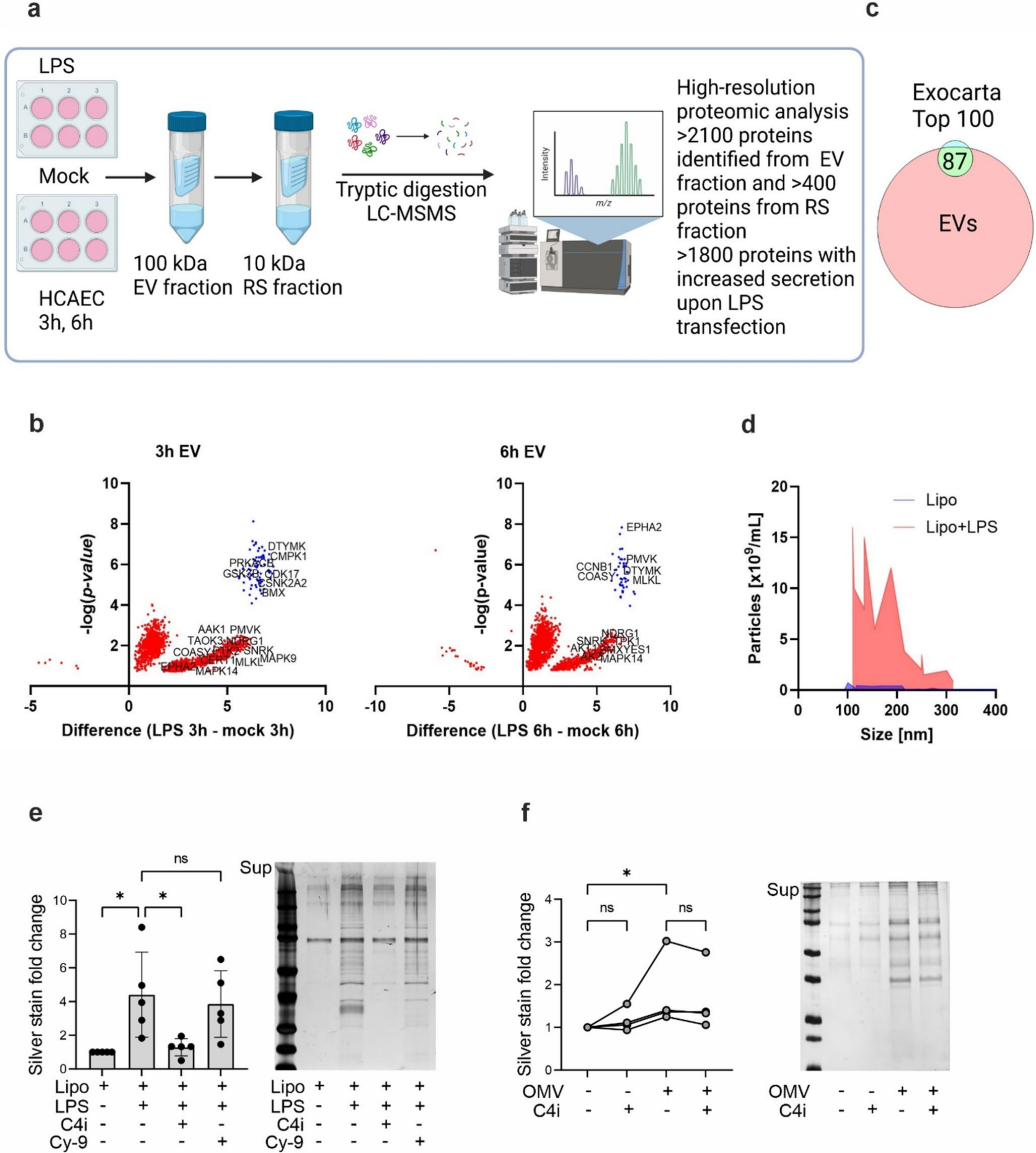
**Noncanonical inflammasome activation induces robust EV-mediated protein secretion in endothelial cells**. **a**) Overview of the proteomics workflow. HCAECs were transfected with a complex of ultra-pure LPS (2 µg/ml) and lipofectamine (5 µl/ml) or mock-treated with lipofectamine (5 µl/ml) for 3 h and 6 h. The supernatants were collected, and the EV-containing fraction was isolated by using a 100 kDa filter. The flow-through was filtered a second time with a 10 kDa cut-off to isolate the rest secretome. The proteomes in EV and RS fractions were analyzed using high-resolution mass spectrometry. **b**) Volcano plot of the proteins significantly different between EVs from mock- and LPS-transfected HCAECs at 3 h and 6 h post transfection. Kinases are labeled within the plots. **c**) Overlap of the HCAEC EV protein identifications with the top 100 EV-associated proteins in ExoCarta. **d**) Nanoparticle-tracking analysis of EVs from mock- and LPS-transfected HCAECs at the 6 h time point. **e**) HCAECs were treated with an inhibitor of caspase-4 Ac-LEVD-CHO (C4i, 25 µM) or NLRP3 inflammasome (Cy-9, 10 µM) for 1 h, and then transfected with a complex of ultra-pure LPS (2 µg/ml) and lipofectamine (5 µl/ml) or mock-treated with lipofectamine (5 µl/ml) for 6 h. Proteins in the EV fraction were separated by SDS-PAGE and visualized with silver staining. Representative SDS-PAGE gel shown. **f**) HCAECs were treated with an inhibitor of caspase-4 Ac-LEVD-CHO (C4i, 25 µM) for 1 h and then stimulated with OMVs (10 µg/mL, 16 h). Proteins in the EV fraction were separated by SDS-PAGE and visualized with silver staining. Representative SDS-PAGE gel shown. Statistics: **e**) *n* = 4, One-way ANOVA followed by Sidak´s multiple comparisons test. **f**) *n* = 4, Friedman test followed by Dunn´s multiple comparisons test

To study the effects of caspase-4/5 activation on EV-mediated secretion from endothelial cells, we used size-exclusion filtering to fractionate the cell culture media from mock-transfected and LPS-transfected human primary endothelial cells into the EV and the rest secretome (RS) fractions, and analyzed them using high-resolution, label-free quantitative proteomics (Fig. 3a). We have previously used a similar workflow and shown that activation of the noncanonical inflammasome in human macrophages results in robust protein secretion [21]. Also HCAECs responded to activation of the noncanonical inflammasome by intracellular LPS with a potent protein secretion in the EV fraction already 3 h post transfection, and the protein secretion further increased towards the 6 h time point (Fig. 3a, b, Table S2, Fig. S2a). LPS transfection significantly induced protein secretion: there were > 1800 proteins whose secretion was increased upon LPS transfection compared to mock transfected samples, and of these, > 200 proteins were exclusively identified from the EV fractions (Fig. 3a, b, Table S2). Proteomic analysis of the RS fractions identified over 400 proteins, and of these, the secretion of approx. 300 proteins increased upon non-canonical inflammasome activation (Table S3, Fig. S2b). When comparing our EV dataset to ExoCarta [33] we identified 87 from the top 100 ExoCarta proteins (Fig. 3c). Nanoparticle tracking assay from the serum-free cell supernatants showed that LPS transfection massively increased EV secretion with roughly similar size distribution compared to mock transfection (Fig. 3d).

Robust protein secretion in the EV fraction was dependent on the activation of the noncanonical inflammasome caspase-4/5 in HCAECs, as the caspase-4/5 inhibitor (Ac-LEVD-CHO), blocked the protein secretion (Fig. 3e). Consistent with that, stimulation with extracellular LPS did not induce protein secretion (Fig. S2c), indicating that LPS internalization is required for EV-mediated protein secretion. A small molecule inhibitor of the NLRP3 inflammasome, Cy-9, did not inhibit the protein secretion, although Cy-9 readily inhibited the NLRP3 inflammasome-dependent IL-1β secretion induced by bacterial toxin nigericin in THP-1 monocytes (Fig. 3e, Fig. S2d), further confirming that NLRP3 inflammasome did not contribute to increased protein secretion in the EV fraction.

Similar to LPS transfection, stimulation of HCAECs with *E. Coli* OMVs increased protein secretion in the EV fraction (Fig. 3f). The OMV-induced protein secretion was to some extent inhibited with caspase-4/5 inhibitor, implying that OMVs increase protein secretion in the EV fraction by mechanisms both dependent and independent of noncanonical inflammasome activation.

Taken together, our results indicate that in human primary endothelial cells, noncanonical inflammasome activation robustly enhances the secretion of EVs and their cargo proteins and additionally induces the release of a broad range of proteins induced upon inflammasome activation. The protein secretion is dependent on caspase-4/5 activation but independent of the NLRP3 inflammasome activation. Importantly, physiologically relevant bacteria-derived OMVs also induce noncanonical inflammasome activation and secretion of proteins in the EV fraction.

### Kinases and kinase-mediated signaling pathways are enriched in EVs after LPS transfection

**Fig. 4:**
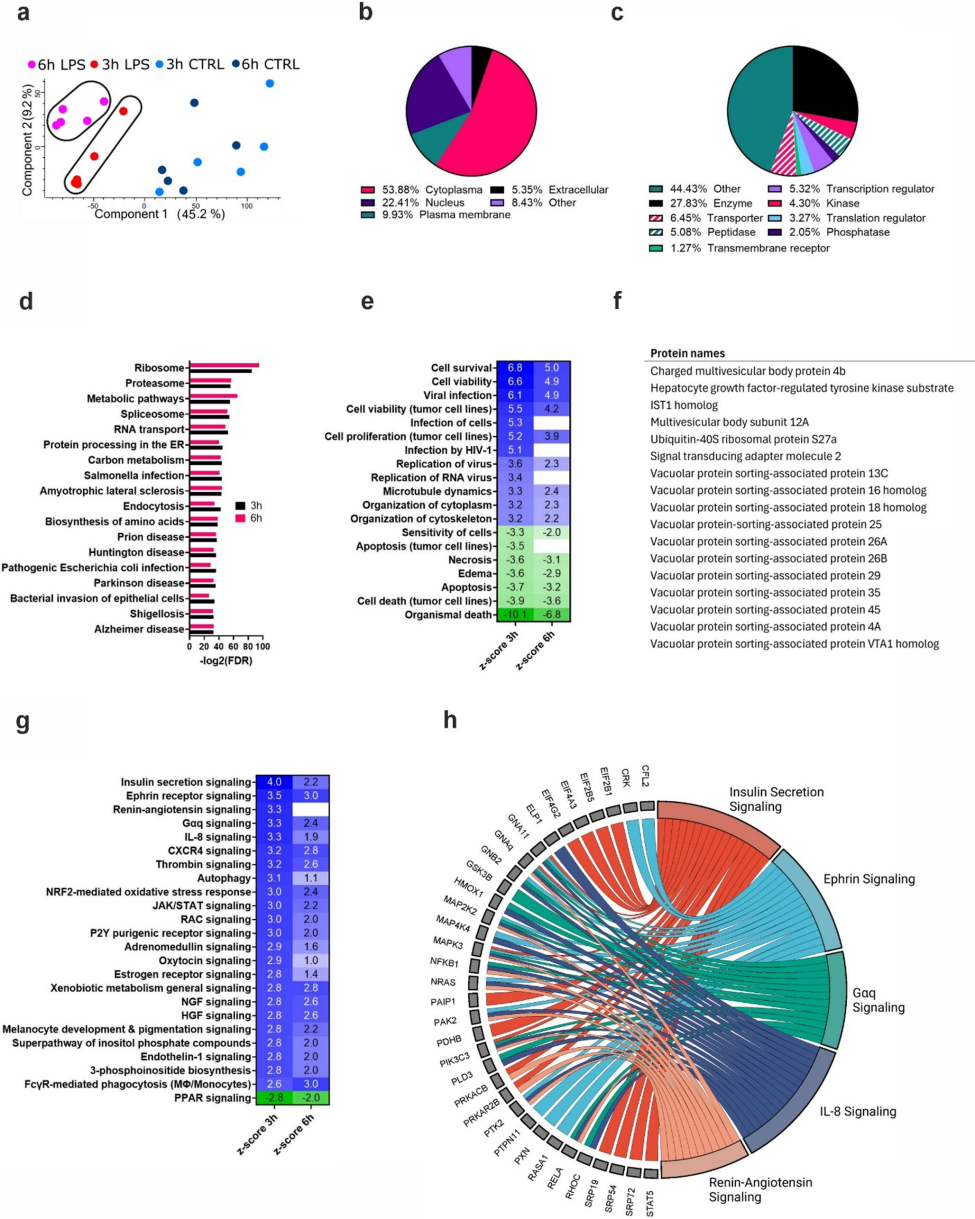
**Noncanonical inflammasome activation substantially increases secretion of biologicallyactive protein species in the extracellular vesicle fraction. a**) Principal component analysis of the EV fraction proteome data. **b**) Subcellular localization of proteins differently enriched in EVs of LPS transfected HCAECs. **c**) Classes of proteins enriched in EVs of LPS transfected HCAECs. **d**) Enriched KEGG terms in the STRING analysis of the EV dataset at 3 h and 6 h. **e**) Comparison analysis in IPA of proteins in EVs at the 3 h and 6 h time points, visualizing the activation z-score in diseases and biological functions. **f**) Proteins identified in the EV dataset that are associated with the ESCRT complex. **g**) Comparison analysis in IPA of proteins in EVs at the 3 h and 6 h time points, visualizing the activation z-score in canonical pathways. **h**) Chord diagram visualizing the 5 most upregulated pathways and the proteins responsible for the enrichment in the 3 h dataset

Principal component analysis of the EV proteome data showed that LPS-transfected samples form distinct groups 3 h and 6 h after stimulation whereas the mock-transfected samples group together at both time points indicating a dynamic change in EV-mediated protein secretion after noncanonical inflammasome activation (Fig. 4a). Classification of the identified proteins in the EV fraction showed that most proteins have cytoplasmic or nuclear annotation (Fig. 4b), and that there is a plethora of biologically active species such as enzymes, kinases, peptidases, phosphatases, as well as regulators of transcription and translation, transporter proteins and transmembrane receptors (Fig. 4c). Interestingly, many kinases were exclusively identified in EV fraction after LPS transfection at both the time points (Fig. 3b, Table S2).

EV fractions were highly enriched in ribosomal proteins and the proteasome component proteins (Fig. S3a, b), as well as proteins involved in several bacterial invasion pathways, such as *Salmonella infection*, *Shigellosis*, *Pathogenic Escherichia coli infection*, and *Bacterial invasion of epithelial cells* (Fig. 4d).

Ingenuity Pathway analysis (IPA) of the EV data showed that in ‘diseases and biological functions’ the most activated terms were related to ‘cell survival’, and terms related to ‘cell death’ were all strongly downregulated at both the time points (Fig. 4e). Also ‘organization of cytoplasm’ and ‘cytoskeleton’ were activated, which is consistent with a state of continuously increased EV secretion. Terms related to ‘viral infection’ were predominantly activated at 3 h, with an increased secretion of nuclear proteins in the vesicles (Fig. 4d, e). Overall, the cells respond to cytosolic LPS by inducing cell survival signaling, which was then observed in proteins secreted in the EV fractions. This may be attributed to the activation of a major membrane-remodeling machinery, the endosomal sorting complex required for transport (ESCRT)-III, which mediates the release of extracellular vesicles from cells with Mixed lineage kinase domain like pseudokinase (MLKL)-induced membrane damage to sustain their viability [34, 35]. Notably, several ESCRT-associated proteins were significantly more secreted in the EV fractions from both the 3 h and 6 h time points after noncanonical inflammasome activation (Fig. 4f, Table S2). Similar to MLKL, the ESCRT machinery is involved in the secretion of EVs in response to NLRP3-induced activation of GSDMD [36]. Recent findings show that GSDMD depends on an adaptor protein IQGAP1 to selectively sort ubiquitinated cargo, particularly IL-1β, into late endosomes. Both GSDMD and IQGAP1 were found to be essential for EV secretion [36]. In line with this, we observed a significant increase in the secretion of IQGAP1 from HCAECs following noncanonical inflammasome activation at both time points (Table S2).

IPA canonical pathway analysis showed that the only strongly downregulated pathway at both the time points is peroxisome proliferator-activated receptor (PPAR) signaling (Fig. 4g). Interestingly, PPARα expression in particular has been shown to be downregulated in sepsis [37]. Upregulated pathways include an array of signaling pathways that are at 3 h largely mediated by mitogen-activated protein kinase 2 (MAP2K2) and mitogen-activated protein kinase 3 (MAPK3), often together with NRAS proto-oncogene, GTPase (NRAS) (Fig. 4h). At the 6 h time point the mitogen activated kinases in these pathways are accompanied by AKT serine/threonine kinase 1 (AKT1) (Fig. S3c).

Taken together, these data show that in the earlier stages of the non-canonical inflammasome activation the cells react more pronouncedly, trying to re-establish homeostasis, while at the later stages of pyroptosis these responses become less pronounced.

### EV-mediated protein secretion is dependent on the pore forming proteins MLKL and GSDMD

**Fig. 5:**
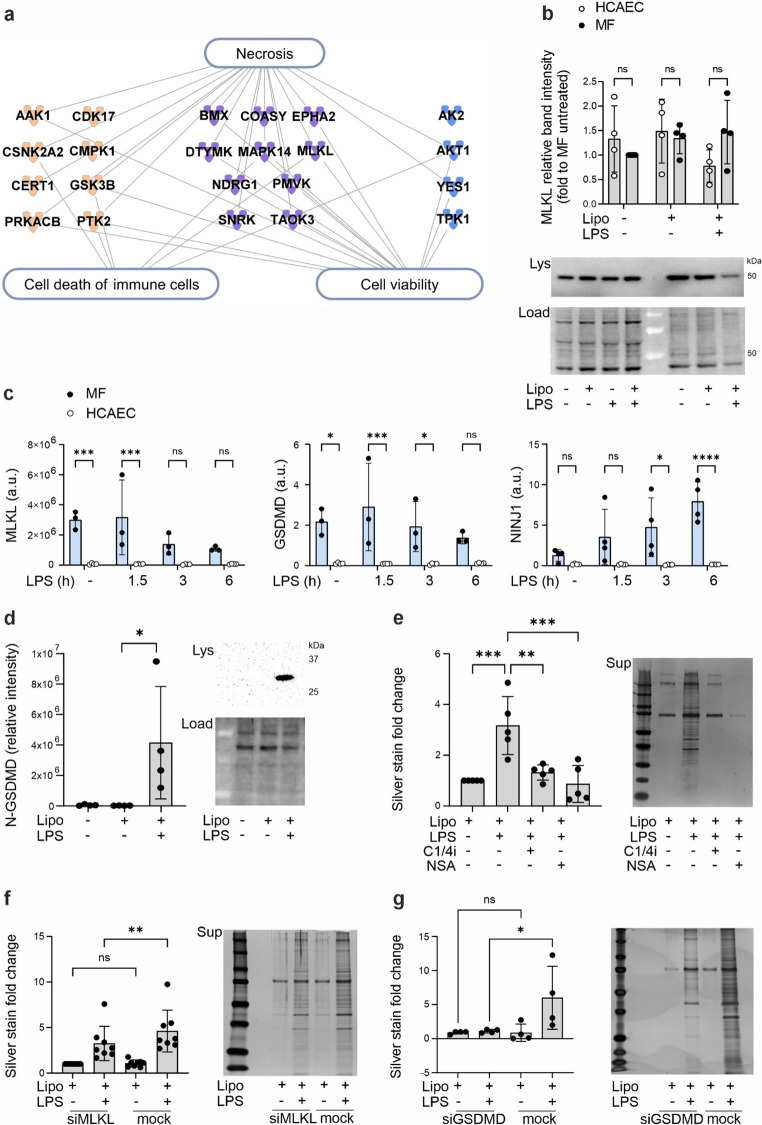
**Protein secretion in the extracellular vesicle fraction is dependent on noncanonical inflammasome activation and partially on endogenous pore forming protein MLKL**. **a**) Kinases associated with terms ‘Necrosis’ and ‘Cell death of immune cell’, and ‘Cell viability’ in Ingenuity Pathway Analysis (IPA) from the proteins that were exclusively identified in EVs of LPS-transfected cells. **b**) HCAECs and human primary macrophages were transfected with a complex of ultra-pure LPS (2 µg/ml) and lipofectamine (5 µl/ml) or mock-treated with lipofectamine (5 µl/ml) for 6 h and the expression of MLKL was blotted. **c**) HCAECs and human primary macrophages were stimulated with extracellular LPS (1 µg/ml) for indicated times and the expression of pyroptosis-related proteins *MLKL*, *GSDMD,* and *NINJ* was detected by RT qPCR. **d)** HCAECs were transfected with a complex of ultra-pure LPS (2 µg/ml) and lipofectamine (5 µl/ml) or mock-treated with lipofectamine (5 µl/ml) for 6 h and the N-terminal active GSDMD (N-GSDMD) was analyzed by Western blotting from cell lysates. Representative Western blots and loading are shown. **e**) HCAECs were treated with an inhibitor of caspase-1/4, Z-YVAD-FMK (C1/4i, 5 µM), or MLKL and GSDMD (NSA, 50 µM) for 1 h and then transfected with a complex of ultra-pure LPS (2 µg/ml) and lipofectamine (5 µl/ml) or mock-treated with lipofectamine (5 µl/ml) for 6 h. Proteins in the EV fraction were separated by SDS-PAGE and visualized with silver staining. Representative SDS-PAGE gel shown. HCAECs were transfected with **(f)** MLKL (30 pmol/ml), **(g)** GSDMD (30 pmol/ml) or mock siRNA (30 pmol/ml) using RNAiMAX (3 µl/ml), and 72 h later noncanonical inflammasome was stimulated by transfection of ultra-pure LPS (LPS 2 µg/ml and/or lipofectamine 5 µl/ml, 6 h). Proteins in the EV fraction were separated by SDS-PAGE and visualized with silver staining. Representative SDS-PAGE gels shown. Statistics: **b**,** c**) two-way ANOVA followed by Sidak´s multiple comparisons test. **d-g**) One-way ANOVA followed by Sidak´s multiple comparisons test. **b-e**,** g**) *n* = 4, **f**) *n* = 8

We observed a major increase in the EV-mediated secretion of kinases related to inflammatory cell death pathways after LPS transfection (Fig. 5a**)**, and proteomic data showed that noncanonical inflammasome activation induced a strong EV-mediated MLKL secretion exclusively in LPS-transfected cells (Table S2). MLKL is primarily known as the terminal executor of necroptosis. Upon activation, it multimerizes and translocates to the cell membrane, forming pores that disrupt cellular integrity, leading to cell swelling, rupture and release of intracellular content. MLKL can mediate pyroptotic cell death independent of GSDMD [38]. Beyond pyroptosis, MLKL plays lesser-known roles in NLRP3 inflammasome activation and release of IL-1β, and importantly, participates in EV generation [39–42]. As robust MLKL secretion was observed only after noncanonical inflammasome activation, we studied its role in EV secretion and cell death. On protein level the expression of MLKL was similar in HCAECs and macrophages (Fig. 5b) but its’ RNA expression was higher in macrophages, similar to expression of *GSDMD* and Ninjurin-1 (*NINJ1)* (Fig. 5c), both of which are mediators of membrane rupture [43]. The presence of serum did not alter the expression of *MLKL* and *GSDMD* in HCAECs (Fig. S4a). GSDMD and Ninj1 mediate cytokine secretion following inflammasome activation from living cells as well as lytic release of cytokines and damage associated molecular patterns from dead cells [44, 45]. Activated N-terminal domain of GSDMD was detected in HCAECs upon LPS transfection (Fig. 5d), indicating that noncanonical inflammasome activation mediates its cleavage. Also another mediator of pyroptosis, Gasdermin E (GSDME) [46], was more abundant in EVs of LPS transfected cells compared to mock-transfected cells (Table S2). GSDME is activated by cleavage by Caspase-3 or 7 [47], and both of them were enriched in EVs of LPS transfected cells (Table S2). *GSDME* was expressed on a rather steady baseline level (average Ct ~ 28) in HCAECs and macrophages (Fig. S4b) and was not induced by noncanonical inflammasome activation in HCAECs (Fig. S4c).

In HCAECs the MLKL and GSDMD inhibitor necrosulfonamide reduced the secretion of proteins in EV fraction to the level of control cells, and its effect was even slightly more pronounced than that observed with caspase-1/4 inhibitor (Z-YVAD-FMK) (Fig. 5e). Also dose dependent reduction of cell death and IL-18 secretion was observed in necrosulfonamide-treated HCAECs and HUVECs (Fig. S5a, b). To further assess the role of MLKL, independent of GSDMD, we silenced *MLKL* expression (Fig. S5c). *MLKL* siRNA significantly reduced protein secretion in the EV fraction after LPS transfection (Fig. 5f). Since the protein release was not completely inhibited by MLKL silencing, we next studied the role of GSDMD. Depletion of GSDMD by siRNA (Fig. S5d) reduced protein secretion in EV fraction following noncanonical inflammasome activation to the level of unstimulated control (Fig. 5g). In conclusion, both MLKL, and even more prominently, GSDMD, contribute to EV release in endothelial cells. Our data confirms the role for MLKL in the protein secretion in EV fraction but suggests that at least GSDMD, and possibly also other proteins, including GSDME or Ninj1, play a role.

### Vesicles secreted from activated HCAECs induce proinflammatory gene expression in human macrophages

**Fig. 6:**
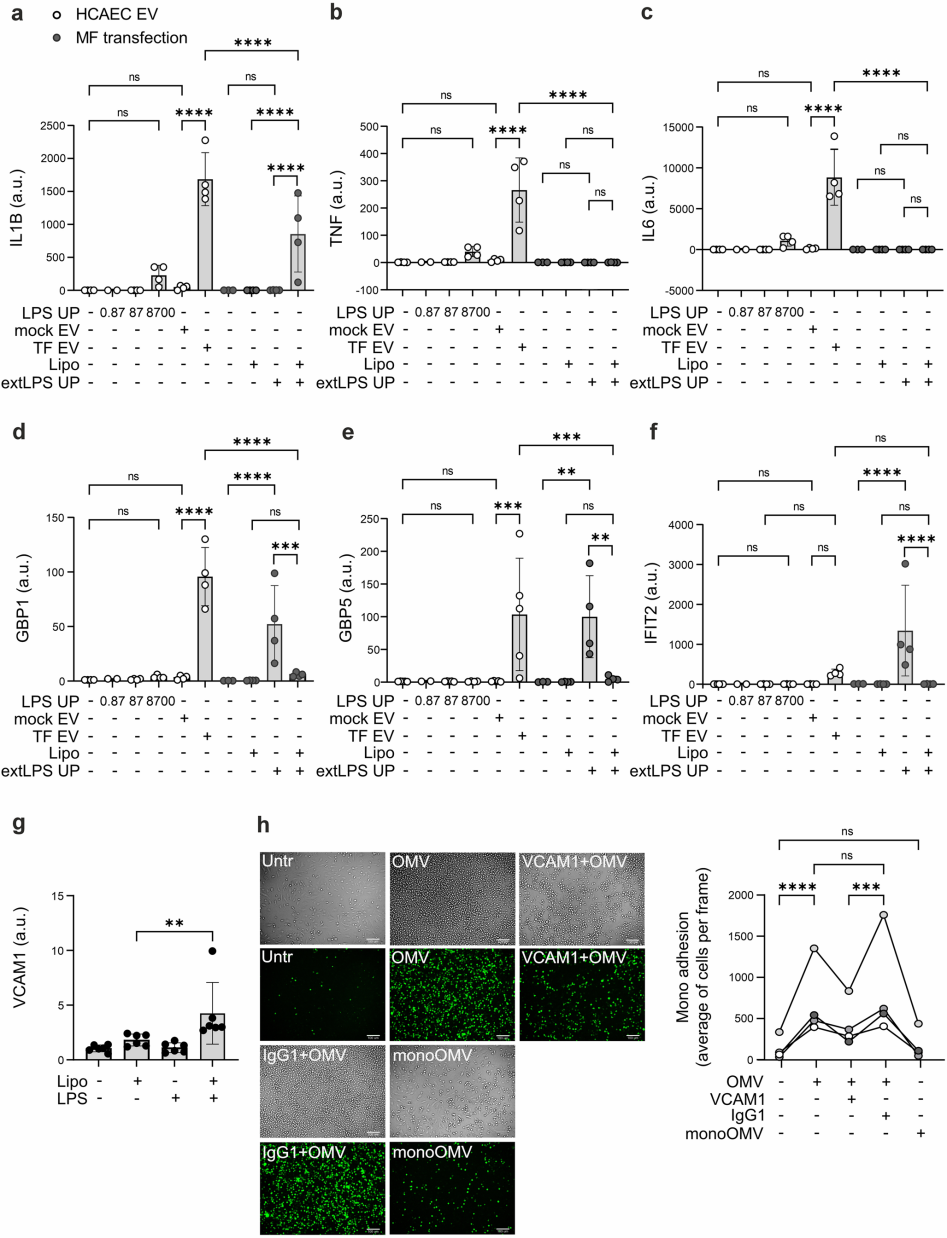
**Noncanonical inflammasome activation increases the adhering of monocytes on endothelial cells and proinflammatory cell-to-cell communication between endothelial cells and macrophages via secretion of extracellular vesicles**. Human primary macrophages were stimulated with HCAEC EVs from LPS- or mock-transfected HCAECs. As a control, human primary macrophages were stimulated with the same amount of ultrapure LPS (0,87 EU) that was detected on EV preparate and with 100x and 10 000x concentrations. Dark grey datapoints: human primary macrophages were stimulated with freshly prepared transfection particles (extracellular ultrapure LPS 2 µg/ml and/or lipofectamine 5 µl/ml, 6 h). Expression of **a**) *IL1B* **b**) *TNF* **(c)** *IL6*, **(d)** *GBP1*, **(e)** *GBP5*, and **(f)** *IFIT2* were analyzed by RT qPCR. **(g)** HCAECs were transfected with a complex of ultra-pure LPS (2 µg/ml) and lipofectamine (5 µl/ml) or mock-treated with lipofectamine (5 µl/ml) for 6 h and the expression of *VCAM* was analyzed by RT qPCR. **(h)** Anti-VCAM-1 antibody or isotype control (IgG1) (both 5 µg/mL) were applied on HCAECs for 2 h, after which cells were activated with OMVs (10 µg/mL) for 2 h and fluorescently labelled (FITC) THP-1 monocytes were let to adhere for 1 h. As a control treatment (monocyte OMV), OMV stimulation was also applied during the adherence period (1 h). Adherent monocytes were detected by fluorescent microscopy, quantifications and representative images shown. Statistics: (**a-g**) One-way ANOVA followed by Sidak´s multiple comparisons test. (**h**) Two-way ANOVA followed by Tukey´s multiple comparisons test. **a-f**) *n* = 4, **g**) *n* = 6, **h**) *n* = 4

To study whether HCAEC-derived EVs could mediate cellular communication and provoke inflammation in the arterial wall, we isolated EVs from LPS and mock transfected HCAECs by ultracentrifugation and used them to stimulate human primary macrophages. Stimulation of macrophages with EVs derived from LPS transfected HCAECs induced robust expression of NF-κB-dependent genes, *IL1B*, tumor necrosis factor (*TNF)*, *IL6*, and interferon stimulated genes, interferon-induced guanylate-binding protein 1 (*GBP1)* and guanylate binding protein 5 (*GBP5)* (Fig. 6a-e) and to a lesser extent also expression of interferon-induced protein with tetratricopeptide repeats 2 (*IFIT2)* (Fig. 6f). No significant induction of gene expression was observed in macrophages stimulated with EVs from mock transfected HCAECs or even with 10 000 times higher concentrations of extracellular ultra-pure LPS than detected in EV preparations, hence observed gene regulation cannot be attributed to LPS carryover in EVs. No cell death was observed in macrophages stimulated with HCAEC-derived EVs (Fig. S6a). Since we could not exclude the possibility that despite washes and extended storage at + 4 °C, EV preparations may still contain some LPS-lipofectamine particles, capable of transfecting the cells, we analyzed the gene expressions from macrophages stimulated with freshly prepared transfection complexes. Transfected ultra-pure LPS induced expression of *IL1B* but none of the other genes studied (Fig. 6a-f). Stimulation of macrophages with the same amount of extracellular ultra-pure LPS than combined with lipofectamine, induced expression of the interferon stimulated genes but none of the NF-κB-dependent cytokines. Differential activation of the macrophages with extracellular ultra-pure LPS and ultra-pure LPS combined with lipofectamine further confirms that vast majority of the LPS combined with lipofectamine is enclosed in liposomes, in which form it is not available for macrophage cell surface receptors.

### Noncanonical inflammasome activation in HCAECs increases monocyte adhesion

Several inflammatory mediators that induce endothelial cell activation or dysfunction increase expression of endothelial adhesion molecules [48]. We studied whether noncanonical inflammasome activation or intracellular LPS induces the expression of the major endothelial cell adhesion molecules, selectins, VCAM1, and intracellular cell adhesion molecule 1 (ICAM1) in HCAECs. LPS transfection significantly increased *VCAM1* gene expression (Fig. 6g) but not expression of *ICAM1*, P-Selectin (*SELP*) or E-Selectin (*SELE*) (Fig. S6b). We further studied adhesion of fluorescently labelled THP-1 monocytes on HCAECs and found that noncanonical inflammasome activation of HCAECs by OMVs or transfected LPS significantly increases THP-1 monocyte adhesion on HCAECs compared to the control treatments, mock transfection or extracellular ultra-pure LPS (Fig. 6h, Fig. S6c). Neutralizing VCAM1 with an antibody reduced the OMV-induced monocyte adhesion, but did not completely block it, suggesting that additional adhesion molecules or other factors contribute (Fig. 6h). To control the possible stimulation of monocytes with LPS-lipofectamine complexes or OMVs despite thorough washes of HCAECs before monocyte coculture, same amounts of transfection particles or OMVs were also added to monocytes for the time of adherence. The controls, LPS loaded lipofectamine particles or OMVs added together with monocytes to the coculture, did not increase adherence, confirming that adhesion was dependent on HCAEC activation (Fig. 6h, Fig. S6c). These results suggest that intracellular LPS can promote monocyte recruitment to the arterial wall and thus further potentiate EV-mediated proinflammatory signaling between endothelial cells and macrophages.

## Discussion

Recent interventional studies have implicated inflammation as one of the key players in the atherosclerotic process [49, 50], but mechanisms which drive the inflammation are less well known. Dysbiosis of intestinal microbiota is more common in patients with atherosclerosis [1, 51]. Dysbiosis is associated with intestinal inflammation and impaired gut barrier function, which lead to increased release of bacterial LPS to systemic circulation. Both intestinal dysbiosis and increased gut permeability have been associated with the progression of ASCVD [1–3]. LPS is a powerful proinflammatory molecule, which can directly promote inflammation by activating immune cells, in particular macrophages in the arterial wall. Here we show that LPS also induces activation of the noncanonical inflammasome in endothelial cells potentially linking the gut microbiota, dysbiosis and increased intestinal permeability with arterial wall inflammation.

We demonstrate that upon entering the cytosol of human primary endothelial cells, LPS induces the activation of noncanonical inflammasome leading to increased IL-18 production and in massive secretion of proteins in the EV-enriched fraction. EVs secreted by endothelial cells in turn activate macrophages to express proinflammatory cytokines. Intracellular LPS also upregulates expression of adhesion molecules, promotes monocyte adhesion to endothelial cells and increases endothelial cell pyroptotic cell death. Endothelial cell damage can also activate the coagulation system, and together all these effects potentially promote vascular inflammation and atherosclerosis. The role of NLRP3 inflammasome in atherosclerosis is well established [49, 52–54], but the role of noncanonical inflammasome is less well known. There is some evidence for the role of noncanonical inflammasome in progression of atherosclerosis [55–59], as for example *CASP4* and *GSDMD* expression levels are upregulated in peripheral blood mononuclear cells of patients with coronary heart disease [60]. Strikingly, hyperlipidemic ApoE−/− mice fed with high-fat high-cholesterol diet exhibited aggravated lesion formation and aortic tissue cell death, which was attenuated by global deletion of caspase-11, suggesting that oxidized phospholipids activate the noncanonical inflammasome, and that caspase-11-GSDMD-axis could be involved in the progression of atherosclerosis [60]. 

We and others have shown that both NLRP3 and noncanonical inflammasome activation induces EV secretion in different cell types [14, 21, 61–64]. In concordance with that, we observed a significant increase in both the numbers of secreted EVs and their protein cargo from HCAECs upon noncanonical inflammasome activation. The secretion of more than 1800 proteins was elevated, with over 200 proteins specifically induced by noncanonical inflammasome activation. In addition, secretion of about 300 proteins was increased by other routes. The proteins secreted in EV fraction included various kinases, regulators of gene expression, transporters, and transmembrane receptors.

The secretome showed enrichment in pathways activated by invasive bacteria, as well as of ribosome and proteasome pathways. Ribosome pathways were also shown to be enriched in EVs of LPS-transfected macrophages [21]. Overall, the EV-mediated protein secretion was independent of extracellular LPS receptor signaling and NLRP3 inflammasome activation in HCAECs. In the endothelial cell line, HUVECs, NLRP3 inflammasome activation likely contributes to EV secretion, and therefore the results obtained from HUVECs may not fully reflect the responses of HCAECs or other primary endothelial cells in the physiological environment. Noncanonical inflammasome activation increased the secretion of proteins associated with cellular stress in the EV proteome, terms related to cellular or oxidative stress, toxicity, immune response, and pathogen-influenced signaling emerged in canonical pathway analysis. Many of the kinases were exclusively secreted in EVs after intracellular LPS detection.

Proteins whose EV-mediated secretion was most prominently increased upon noncanonical inflammasome activation were associated with processes promoting cell survival and cell viability. Conversely, the secretion of proteins related to cell death, including apoptosis and necrosis, were strongly downregulated. Notably, simultaneously to secretion of cell survival pathway proteins, the secretion of ESCRT-associated proteins was induced. The ESCRT-III machinery is essential to exosome and ectosome secretion, but it has also been proposed to delay membrane damage-induced cell death by excising the damaged parts of the cell membrane, thereby providing dying cells more time to signal their surroundings [34, 35, 39, 65]. However, the net effect of noncanonical inflammasome activation on cell viability was a modest increase in LDH secretion in HCAECs and HUVECs, indicating the onset of inflammatory cell death. The cell death was initiated already by three hours post transfection and it progressively increased towards six hour time point. Maintaining the integrity of endothelium is crucial particularly in coronary arteries, and therefore the pyroptotic cell death of endothelial cells can be highly detrimental and thus, this may explain the initial induction of survival pathways. It is conceivable that ESCRT machinery was involved in extracellular vesicle formation in HCAECs as suggested by the release of pore forming proteins and newly found adaptor protein IQGAP1 [36], it is also possible that ESCRT signaling helped limit cell death, contributing to the relatively modest induction of cell death in these critical cells.

During noncanonical inflammasome activation, formation of GSDMD, GSDME, or MLKL pores permeabilize the cells leading to cell death [66, 67]. They further mediate the secretion of proinflammatory mediators and alarmins either as captured inside vesicles or as soluble proteins released upon cell lysis [29]. We observed a high EV-mediated secretion of MLKL in HCAECs only after activation with intracellular LPS. Also GSDME, along with its activating caspases 3 and 7, were enriched in EVs derived from LPS transfected cells. As ESCRT-III machinery repairs the cell membrane by excising pore containing parts of cell membrane and removes the damaged membrane parts by vesicle secretion [39, 68], the increased secretion of MLKL from HCAECs likely serves to limit the cell death. Consistent with previous findings, depletion and chemical inhibition of MLKL and GSDMD both reduced total protein secretion in the EV-enriched fraction, suggesting a similar role for MLKL in HCAECs than in dendritic cells [39, 69]. The efficient inhibition of EV secretion upon depletion of GSDMD suggests that in HCAECs GSDMD plays a central role in vesicle release, with MLKL acting as a supporting factor. It has also been shown that, particularly under inflammatory conditions, pyroptotic EVs loaded with GSDMD pores can amplify cell death by transferring these pores to neighboring non-stimulated cells [70]. This mechanism may contribute to progression of conditions such as ASCVD and sepsis.

Endothelial cell-derived EVs have been found in atherosclerotic lesions, where they constitute about 8% of the total EVs [71]. Endothelial- and platelet-derived EVs have also been suggested as independent biomarkers for coronary artery disease [72]. The in vivo mouse studies suggest a role for the noncanonical inflammasome in monocyte infiltration. In mice, inhibition of noncanonical inflammasome activation by global or endothelial cell specific deletion of caspase-11, reduced infiltration of monocytes to arterial intima and lung microvessel leakiness, respectively [14, 60]. We observed that in HCAECs, noncanonical inflammasome activation increased significantly the expression of *VCAM1*, thus potentially enhancing immune cell influx. Both transfected LPS and OMVs promoted monocyte adherence on HCAECs and inhibition of VCAM1 reduced the adhesion. The increased adhesion molecule expression upon noncanonical inflammasome activation may represent yet another necroptosis-independent function of MLKL. Dai et al. demonstrated that TNF-induced MLKL activation stabilized *VCAM1*, *ICAM1*, and E-selectin transcripts, which promoted leukocyte adhesion on HUVECs. Consistently, MLKL deficiency reduced TNF-induced leukocyte adhesion to endothelial cells in vivo [73]. Purified EVs from HCAECs activated with intracellular LPS induced transcription of a set of proinflammatory NF-κB-dependent genes and interferon stimulated genes in human primary macrophages, implying that noncanonical inflammasome activation in endothelial cells could provoke proinflammatory responses in the subendothelial tissue cells.

To conclude, during metabolic endotoxemia circulating LPS can access endothelial cells either within OMVs, as bound to EVs or as bound to an endogenous carrier molecule, such as HMGB1, and activate noncanonical inflammasome. In the endothelium the noncanonical inflammasome activation causes endothelial damage, promotes monocyte recruitment by upregulating *VCAM1*, and enhances the secretion and protein content of EVs, which can further lead to enhanced leukocyte infiltration and proinflammatory activation of macrophages in arterial intima. Therefore, activation of noncanonical inflammasome in endothelial cells represents a plausible link between intestinal dysbiosis, increased intestinal permeability, metabolic endotoxemia and arterial wall inflammation.

## Supplementary Information

Below is the link to the electronic supplementary material.

Supplementary Material 1 Table S1 (PDF 27.3 MB )

Supplementary Material 2 (PDF 6.18 MB )

Supplementary Material 3 Table S2

Supplementary Material 4 Table S3

## Data Availability

Cell lines used in this study are commercially available and this study did not generate new unique reagents; all reagents are commercially available.

## Abbreviations

AKT1: AKT serine/threonine kinase 1
ASCVD: Atherosclerotic cardiovascular diseases
CCL2: Chemokine (C-C motif) ligand 2
ESCRT: Endosomal sorting complex required for transport
EV: Extracellular vesicle
GBP1: Interferon-induced guanylate-binding protein 1
GBP5: Guanylate binding protein 5
GSDMD: Gasdermin D
GSDME: Gasdermin E
HCAECs: Human coronary artery endothelial cells
HMGB1: High mobility group box 1
HUVECs: Human umbilical vein endothelial cells
ICAM1: Intracellular cell adhesion molecule 1
IFIT2: Interferon-induced protein with tetratricopeptide repeats 2
IL: Interleukin
LDH: Lactate dehydrogenase
LPS: Lipopolysaccharide
MAP2K2: Mitogen-activated protein kinase 2
MAPK3: Mitogen-activated protein kinase 3
MLKL: Mixed lineage kinase domain like pseudokinase
NINJ1: Ninjurin-1
NLRP3: Nucleotide-binding oligomerization domain, leucine rich repeat-containing protein 3
NRAS: NRAS proto-oncogene, GTPase
OMV: Outer membrane vesicle
PPAR: Peroxisome proliferator-activated receptor
RS: Rest secretome
TLR: Toll-like receptor
TNF: Tumor necrosis factor
VCAM1: Vascular cell adhesion molecule 1

## Data and code availability

The original blots are presented in the Source data file. The mass spectrometry-based proteomics data have been deposited to the ProteomeXchange Consortium via the PRIDE [74] partner repository with the dataset identifier PXD062376.

This publication did not generate original code or structures. Used software packages are listed with citations in Materials and Methods. Additional information required to reanalyze the data, including microscopy data and RNA expression data reported in this paper is available from the lead contact (Katariina.nurmi@helsinki.fi) upon request. Data access will be considered for research purposes and non-commercial use only.
