# Supplementary material for: Lipopolysaccharides Drive Proinflammatory Extracellular Vesicle Secretion in Coronary Artery Endothelial Cells via Noncanonical Inflammasome Activation
Source: Cell Mol Life Sci. 2026 Apr 21;83(1):239. doi: 10.1007/s00018-025-06006-y (PMC13237317; doi:10.1007/s00018-025-06006-y)

Supplementary Table 1

Quantitative Real-time PCR primers.

Gene	FW	REV
IL1B	TGGCAATGAGGATGACTTGT	GGAAAGAAGGTGCTCAGGTC
NLRP3	CAACTGCAACCTCACGTCAC	ACGGTCAGCTCAGGCTTTTC
IL18	TCAACTCTCTCCTGTGAGAACAAA	GTCCTGGGACACTTCTCTGAAA
CASP4	TTGAAAATGGAAGCCACAAGCA	CAAGCTGTACTAATGAAGGTGCTC
CASP5	CTTCAAGGCCTGGGCTACAC	TCAGCACTGACTCCATATCCCTG
IL6	AGGAGACTTGCCTGGTGAAA	GAGGTGCCCATGCTACATTT
CCL2	GATCTCAGTGCAGAGGCTCG	TCTCCTTGGCCACAATGGTC
GSDMD	GGCCCAGCTGGTTATTGACT	AGGGACCCCATCTGTCAGGA
TNF	TGCTGCACTTTGGAGTGATCG	ATCTCTCAGCTCCACGCCATT
GBP1	GGAGATTGAAGTGGAACGTGTGA	TCTGCTTTCTTTTTGAAATCCCTCT
GBP5	GTACTATCGGGAGCCTCGGA	TTTGAATCGCCGCCAACCTT
IFIT2	GCCGAACAGCTGAGAATTGC	AGGCCAGTAGGTTGCACATTG
GSDME	GTTTGCCAAAGCAACCAGGA	TGAGTACATCGCCAAGGGTG
MLKL	GTGGAAGAGCGAGCTTTTTGG	TTTCCTTGGTCCTGGAGCATC
RPLP0	GAAATCCTGAGTGATGTGCAGC	TCGAACACCTGCTGGATGAC

Supplementary File

CellProfiler 4.2.8 Image analysis pipeline.

{
"has_image_plane_details": false,
"date_revision": 428,
"module_count": 14,
"modules": [
{
"attributes": {
"module_num": 1,
"notes": [
"To begin creating your project, use the Images module to compile a list of files and/or folders that you want to analyze. You can also specify a set of rules to include only the desired files in your selected folders."
],
"show_window": false,
"wants_pause": false,
"svn_version": "Unknown",
"enabled": true,
"variable_revision_number": 2,
"batch_state": "array([], dtype=uint8)",
"module_name": "Images",
"module_path": "cellprofiler_core.modules.images.Images"
},
"settings": [
{
"name": "cellprofiler_core.setting._path_list_display.PathListDisplay",
"text": "",
"value": ""
},
{
"name": "cellprofiler_core.setting.choice._choice.Choice",
"text": "Filter images?",
"value": "Images only"
},
{
"name": "cellprofiler_core.setting.filter._filter.Filter",
"text": "Select the rule criteria",
"value": "and (extension does isimage) (directory doesnot containregexp \"[\\\\\\\\/]\\\\.\")"
}
]
},
{
"attributes": {
"module_num": 2,
"notes": [
"The Metadata module optionally allows you to extract information describing your images (i.e, metadata) which will be stored along with your measurements. This information can be contained in the file name and/or location, or in an external file."
],
"show_window": false,
"wants_pause": false,
"svn_version": "Unknown",
"enabled": true,

```
            "variable_revision_number": 6,
            "batch_state": "array([], dtype=uint8)",
            "module_name": "Metadata",
            "module_path": "cellprofiler_core.modules.metadata.Metadata"
        },
        "settings": [
            {
                "name": "cellprofiler_core.setting._binary.Binary",
                "text": "Extract metadata?",
                "value": "No"
            },
            {
                "name": "cellprofiler_core.setting.choice._choice.Choice",
                "text": "Metadata data type",
                "value": "Text"
            },
            {
                "name": "cellprofiler_core.setting._data_types.DataTypes",
                "text": "Metadata types",
                "value": "{}"
            },
            {
                "name": "cellprofiler_core.setting._hidden_count.HiddenCount",
                "text": "Extraction method count",
                "value": "1"
            },
            {
                "name": "cellprofiler_core.setting.choice._choice.Choice",
                "text": "Metadata extraction method",
                "value": "Extract from file/folder names"
            },
            {
                "name": "cellprofiler_core.setting.choice._choice.Choice",
                "text": "Metadata source",
                "value": "File name"
            },
            {
                "name": "cellprofiler_core.setting._regexp_text.RegexpText",
                "text": "Regular expression to extract from file name",
                "value": "^(?P<Plate>.*)_(?P<Well>[A-P][0-9]{2})_s(?P<Site>[0-
9])_w(?P<ChannelNumber>[0-9])"
            },
            {
                "name": "cellprofiler_core.setting._regexp_text.RegexpText",
                "text": "Regular expression to extract from folder name",
                "value": "(?P<Date>[0-9]{4}_[0-9]{2}_[0-9]{2})$"
            },
            {
                "name": "cellprofiler_core.setting.choice._choice.Choice",
                "text": "Extract metadata from",
                "value": "All images"
            },
            {
                "name": "cellprofiler_core.setting.filter._filter.Filter",
                "text": "Select the filtering criteria",
                "value": "and (file does contain \"\")"
            },
```

{
"name": "cellprofiler_core.setting.text._directory.Directory",
"text": "Metadata file location",
"value": "Elsewhere...|"
},
{
"name": "cellprofiler_core.setting._joiner.Joiner",
"text": "Match file and image metadata",
"value": "[]"
},
{
"name": "cellprofiler_core.setting._binary.Binary",
"text": "Use case insensitive matching?",
"value": "No"
},
{
"name": "cellprofiler_core.setting.text._filename.Filename",
"text": "Metadata file name",
"value": "None"
},
{
"name": "cellprofiler_core.setting._binary.Binary",
"text": "Does cached metadata exist?",
"value": "No"
}
]
},
{
"attributes": {
"module_num": 3,
"notes": [
"The NamesAndTypes module allows you to assign a meaningful name to each image by which other modules will refer to it."
],
"show_window": false,
"wants_pause": false,
"svn_version": "Unknown",
"enabled": true,
"variable_revision_number": 8,
"batch_state": "array([], dtype=uint8)",
"module_name": "NamesAndTypes",
"module_path": "cellprofiler_core.modules.namesandtypes.NamesAndTypes"
},
"settings": [
{
"name": "cellprofiler_core.setting.choice._choice.Choice",
"text": "Assign a name to",
"value": "All images"
},
{
"name": "cellprofiler_core.setting.choice._choice.Choice",
"text": "Select the image type",
"value": "Grayscale image"
},
{
"name":
"cellprofiler_core.setting.text.alphanumeric.name.image_name._file_image_name.FileImageName",

"text": "Name to assign these images",
"value": "DNA"
},
{
"name": "cellprofiler_core.setting._joiner.Joiner",
"text": "Match metadata",
"value": "[]"
},
{
"name": "cellprofiler_core.setting.choice._choice.Choice",
"text": "Image set matching method",
"value": "Order"
},
{
"name": "cellprofiler_core.setting.choice._choice.Choice",
"text": "Set intensity range from",
"value": "Image metadata"
},
{
"name": "cellprofiler_core.setting._hidden_count.HiddenCount",
"text": "Assignments count",
"value": "1"
},
{
"name": "cellprofiler_core.setting._hidden_count.HiddenCount",
"text": "Single images count",
"value": "0"
},
{
"name": "cellprofiler_core.setting.text.number._float.Float",
"text": "Maximum intensity",
"value": "255.0"
},
{
"name": "cellprofiler_core.setting._binary.Binary",
"text": "Process as 3D?",
"value": "No"
},
{
"name": "cellprofiler_core.setting.text.number._float.Float",
"text": "Relative pixel spacing in X",
"value": "1.0"
},
{
"name": "cellprofiler_core.setting.text.number._float.Float",
"text": "Relative pixel spacing in Y",
"value": "1.0"
},
{
"name": "cellprofiler_core.setting.text.number._float.Float",
"text": "Relative pixel spacing in Z",
"value": "1.0"
},
{
"name": "cellprofiler_core.setting.filter._filter.Filter",
"text": "Select the rule criteria",
"value": "and (file does contain \"\")"

},
{
"name":
"cellprofiler_core.setting.text.alphanumeric.name.image_name._file_image_name.FileImageName",
"text": "Name to assign these images",
"value": "DNA"
},
{
"name": "cellprofiler_core.setting.text.alphanumeric.name._label_name.LabelName",
"text": "Name to assign these objects",
"value": "Cell"
},
{
"name": "cellprofiler_core.setting.choice._choice.Choice",
"text": "Select the image type",
"value": "Grayscale image"
},
{
"name": "cellprofiler_core.setting.choice._choice.Choice",
"text": "Set intensity range from",
"value": "Image metadata"
},
{
"name": "cellprofiler_core.setting.text.number._float.Float",
"text": "Maximum intensity",
"value": "255.0"
}
]
},
{
"attributes": {
"module_num": 4,
"notes": [
"The Groups module optionally allows you to split your list of images into image subsets (groups) which will be processed independently of each other. Examples of groupings include screening batches, microtiter plates, time-lapse movies, etc."
],
"show_window": false,
"wants_pause": false,
"svn_version": "Unknown",
"enabled": true,
"variable_revision_number": 2,
"batch_state": "array([], dtype=uint8)",
"module_name": "Groups",
"module_path": "cellprofiler_core.modules.groups.Groups"
},
"settings": [
{
"name": "cellprofiler_core.setting._binary.Binary",
"text": "Do you want to group your images?",
"value": "No"
},
{
"name": "cellprofiler_core.setting._hidden_count.HiddenCount",
"text": "grouping metadata count",
"value": "1"
},

```
            {
               "name": "cellprofiler_core.setting.choice._choice.Choice",
               "text": "Metadata category",
               "value": "None"
            }
         ]
      },
      {
         "attributes": {
            "module_num": 5,
            "notes": [],
            "show_window": true,
            "wants_pause": false,
            "svn_version": "Unknown",
            "enabled": true,
            "variable_revision_number": 2,
            "batch_state": "array([], dtype=uint8)",
            "module_name": "CorrectIlluminationCalculate",
            "module_path": "cellprofiler.modules.correctilluminationcalculate.CorrectIlluminationCalculate"
         },
         "settings": [
            {
               "name":
"cellprofiler_core.setting.subscriber.image_subscriber._image_subscriber.ImageSubscriber",
               "text": "Select the input image",
               "value": "DNA"
            },
            {
               "name":
"cellprofiler_core.setting.text.alphanumeric.name.image_name._image_name.ImageName",
               "text": "Name the output image",
               "value": "IllumBlue"
            },
            {
               "name": "cellprofiler_core.setting.choice._choice.Choice",
               "text": "Select how the illumination function is calculated",
               "value": "Regular"
            },
            {
               "name": "cellprofiler_core.setting._binary.Binary",
               "text": "Dilate objects in the final averaged image?",
               "value": "No"
            },
            {
               "name": "cellprofiler_core.setting.text.number.integer._integer.Integer",
               "text": "Dilation radius",
               "value": "1"
            },
            {
               "name": "cellprofiler_core.setting.text.number.integer._integer.Integer",
               "text": "Block size",
               "value": "60"
            },
            {
               "name": "cellprofiler_core.setting.choice._choice.Choice",
               "text": "Rescale the illumination function?",
               "value": "Yes"
```

},
{
"name": "cellprofiler_core.setting.choice._choice.Choice",
"text": "Calculate function for each image individually, or based on all images?",
"value": "Each"
},
{
"name": "cellprofiler_core.setting.choice._choice.Choice",
"text": "Smoothing method",
"value": "Fit Polynomial"
},
{
"name": "cellprofiler_core.setting.choice._choice.Choice",
"text": "Method to calculate smoothing filter size",
"value": "Automatic"
},
{
"name": "cellprofiler_core.setting.text.number.integer._integer.Integer",
"text": "Approximate object diameter",
"value": "10"
},
{
"name": "cellprofiler_core.setting.text.number.integer._integer.Integer",
"text": "Smoothing filter size",
"value": "10"
},
{
"name": "cellprofiler_core.setting._binary.Binary",
"text": "Retain the averaged image?",
"value": "No"
},
{
"name":
"cellprofiler_core.setting.text.alphanumeric.name.image_name._image_name.ImageName",
"text": "Name the averaged image",
"value": "IllumBlueAvg"
},
{
"name": "cellprofiler_core.setting._binary.Binary",
"text": "Retain the dilated image?",
"value": "No"
},
{
"name":
"cellprofiler_core.setting.text.alphanumeric.name.image_name._image_name.ImageName",
"text": "Name the dilated image",
"value": "IllumBlueDilated"
},
{
"name": "cellprofiler_core.setting._binary.Binary",
"text": "Automatically calculate spline parameters?",
"value": "Yes"
},
{
"name": "cellprofiler_core.setting.choice._choice.Choice",
"text": "Background mode",
"value": "auto"

},
{
"name": "cellprofiler_core.setting.text.number.integer._integer.Integer",
"text": "Number of spline points",
"value": "5"
},
{
"name": "cellprofiler_core.setting.text.number._float.Float",
"text": "Background threshold",
"value": "2.0"
},
{
"name": "cellprofiler_core.setting.text.number._float.Float",
"text": "Image resampling factor",
"value": "2.0"
},
{
"name": "cellprofiler_core.setting.text.number.integer._integer.Integer",
"text": "Maximum number of iterations",
"value": "40"
},
{
"name": "cellprofiler_core.setting.text.number._float.Float",
"text": "Residual value for convergence",
"value": "0.001"
}
]
},
{
"attributes": {
"module_num": 6,
"notes": [],
"show_window": true,
"wants_pause": false,
"svn_version": "Unknown",
"enabled": true,
"variable_revision_number": 5,
"batch_state": "array([], dtype=uint8)",
"module_name": "CorrectIlluminationApply",
"module_path": "cellprofiler.modules.correctilluminationapply.CorrectIlluminationApply"
},
"settings": [
{
"name":
"cellprofiler_core.setting.subscriber.image_subscriber._image_subscriber.ImageSubscriber",
"text": "Select the input image",
"value": "DNA"
},
{
"name":
"cellprofiler_core.setting.text.alphanumeric.name.image_name._image_name.ImageName",
"text": "Name the output image",
"value": "CorrBlue"
},
{
"name":
"cellprofiler_core.setting.subscriber.image_subscriber._image_subscriber.ImageSubscriber",

"text": "Select the illumination function",
"value": "IllumBlue"
},
{
"name": "cellprofiler_core.setting.choice._choice.Choice",
"text": "Select how the illumination function is applied",
"value": "Divide"
},
{
"name": "cellprofiler_core.setting._binary.Binary",
"text": "Set output image values less than 0 equal to 0?",
"value": "Yes"
},
{
"name": "cellprofiler_core.setting._binary.Binary",
"text": "Set output image values greater than 1 equal to 1?",
"value": "Yes"
}
]
},
{
"attributes": {
"module_num": 7,
"notes": [],
"show_window": true,
"wants_pause": false,
"svn_version": "Unknown",
"enabled": true,
"variable_revision_number": 15,
"batch_state": "array([], dtype=uint8)",
"module_name": "IdentifyPrimaryObjects",
"module_path": "cellprofiler.modules.identifyprimaryobjects.IdentifyPrimaryObjects"
},
"settings": [
{
"name":
"cellprofiler_core.setting.subscriber.image_subscriber._image_subscriber.ImageSubscriber",
"text": "Select the input image",
"value": "CorrBlue"
},
{
"name": "cellprofiler_core.setting.text.alphanumeric.name._label_name.LabelName",
"text": "Name the primary objects to be identified",
"value": "IdentifyPrimaryObjects"
},
{
"name": "cellprofiler_core.setting.range.integer_range._integer_range.IntegerRange",
"text": "Typical diameter of objects, in pixel units (Min,Max)",
"value": "15,60"
},
{
"name": "cellprofiler_core.setting._binary.Binary",
"text": "Discard objects outside the diameter range?",
"value": "Yes"
},
{
"name": "cellprofiler_core.setting._binary.Binary",

```
        "text": "Discard objects touching the border of the image?",
        "value": "No"
    },
    {
        "name": "cellprofiler_core.setting.choice._choice.Choice",
        "text": "Method to distinguish clumped objects",
        "value": "Intensity"
    },
    {
        "name": "cellprofiler_core.setting.choice._choice.Choice",
        "text": "Method to draw dividing lines between clumped objects",
        "value": "Intensity"
    },
    {
        "name": "cellprofiler_core.setting.text.number.integer._integer.Integer",
        "text": "Size of smoothing filter",
        "value": "10"
    },
    {
        "name": "cellprofiler_core.setting.text.number._float.Float",
        "text": "Suppress local maxima that are closer than this minimum allowed distance",
        "value": "7.0"
    },
    {
        "name": "cellprofiler_core.setting._binary.Binary",
        "text": "Speed up by using lower-resolution image to find local maxima?",
        "value": "Yes"
    },
    {
        "name": "cellprofiler_core.setting.choice._choice.Choice",
        "text": "Fill holes in identified objects?",
        "value": "After declumping only"
    },
    {
        "name": "cellprofiler_core.setting._binary.Binary",
        "text": "Automatically calculate size of smoothing filter for declumping?",
        "value": "Yes"
    },
    {
        "name": "cellprofiler_core.setting._binary.Binary",
        "text": "Automatically calculate minimum allowed distance between local maxima?",
        "value": "Yes"
    },
    {
        "name": "cellprofiler_core.setting.choice._choice.Choice",
        "text": "Handling of objects if excessive number of objects identified",
        "value": "Continue"
    },
    {
        "name": "cellprofiler_core.setting.text.number.integer._integer.Integer",
        "text": "Maximum number of objects",
        "value": "500"
    },
    {
        "name": "cellprofiler_core.setting._binary.Binary",
        "text": "Use advanced settings?",
        "value": "Yes"
```

```
},
{
    "name": "cellprofiler_core.setting.text.number.integer._integer.Integer",
    "text": "Threshold setting version",
    "value": "12"
},
{
    "name": "cellprofiler_core.setting.choice._choice.Choice",
    "text": "Threshold strategy",
    "value": "Global"
},
{
    "name": "cellprofiler_core.setting.choice._choice.Choice",
    "text": "Thresholding method",
    "value": "Otsu"
},
{
    "name": "cellprofiler_core.setting.text.number._float.Float",
    "text": "Threshold smoothing scale",
    "value": "1.3488"
},
{
    "name": "cellprofiler_core.setting.text.number._float.Float",
    "text": "Threshold correction factor",
    "value": "1.0"
},
{
    "name": "cellprofiler_core.setting.range._float_range.FloatRange",
    "text": "Lower and upper bounds on threshold",
    "value": "0.1,1.0"
},
{
    "name": "cellprofiler_core.setting.text.number._float.Float",
    "text": "Manual threshold",
    "value": "0.0"
},
{
    "name": "cellprofiler_core.setting._measurement.Measurement",
    "text": "Select the measurement to threshold with",
    "value": "None"
},
{
    "name": "cellprofiler_core.setting.choice._choice.Choice",
    "text": "Two-class or three-class thresholding?",
    "value": "Three classes"
},
{
    "name": "cellprofiler_core.setting._binary.Binary",
    "text": "Log transform before thresholding?",
    "value": "Yes"
},
{
    "name": "cellprofiler_core.setting.choice._choice.Choice",
    "text": "Assign pixels in the middle intensity class to the foreground or the background?",
    "value": "Foreground"
},
{
```

```
            "name": "cellprofiler_core.setting.text.number.integer._integer.Integer",
            "text": "Size of adaptive window",
            "value": "50"
          },
          {
            "name": "cellprofiler_core.setting.text.number._float.Float",
            "text": "Lower outlier fraction",
            "value": "0.05"
          },
          {
            "name": "cellprofiler_core.setting.text.number._float.Float",
            "text": "Upper outlier fraction",
            "value": "0.05"
          },
          {
            "name": "cellprofiler_core.setting.choice._choice.Choice",
            "text": "Averaging method",
            "value": "Mean"
          },
          {
            "name": "cellprofiler_core.setting.choice._choice.Choice",
            "text": "Variance method",
            "value": "Standard deviation"
          },
          {
            "name": "cellprofiler_core.setting.text.number._float.Float",
            "text": "# of deviations",
            "value": "2.0"
          },
          {
            "name": "cellprofiler_core.setting.choice._choice.Choice",
            "text": "Thresholding method",
            "value": "Minimum Cross-Entropy"
          }
        ]
      },
      {
        "attributes": {
          "module_num": 8,
          "notes": [],
          "show_window": true,
          "wants_pause": true,
          "svn_version": "Unknown",
          "enabled": true,
          "variable_revision_number": 3,
          "batch_state": "array([], dtype=uint8)",
          "module_name": "MeasureObjectSizeShape",
          "module_path": "cellprofiler.modules.measureobjectsizeshape.MeasureObjectSizeShape"
        },
        "settings": [
          {
            "name":
"cellprofiler_core.setting.subscriber.list_subscriber._label_list_subscriber.LabelListSubscriber",
            "text": "Select object sets to measure",
            "value": "IdentifyPrimaryObjects"
          },
          {
```

```
            "name": "cellprofiler_core.setting._binary.Binary",
            "text": "Calculate the Zernike features?",
            "value": "Yes"
          },
          {
            "name": "cellprofiler_core.setting._binary.Binary",
            "text": "Calculate the advanced features?",
            "value": "Yes"
          }
        ]
      },
      {
        "attributes": {
          "module_num": 9,
          "notes": [],
          "show_window": true,
          "wants_pause": false,
          "svn_version": "Unknown",
          "enabled": true,
          "variable_revision_number": 10,
          "batch_state": "array([], dtype=uint8)",
          "module_name": "FilterObjects",
          "module_path": "cellprofiler.modules.filterobjects.FilterObjects"
        },
        "settings": [
          {
            "name": "cellprofiler_core.setting.subscriber._label_subscriber.LabelSubscriber",
            "text": "Select the objects to filter",
            "value": "IdentifyPrimaryObjects"
          },
          {
            "name": "cellprofiler_core.setting.text.alphanumeric.name._label_name.LabelName",
            "text": "Name the output objects",
            "value": "FilterObjects"
          },
          {
            "name": "cellprofiler_core.setting.choice._choice.Choice",
            "text": "Select the filtering mode",
            "value": "Measurements"
          },
          {
            "name": "cellprofiler_core.setting.choice._choice.Choice",
            "text": "Select the filtering method",
            "value": "Limits"
          },
          {
            "name": "cellprofiler_core.setting.subscriber._label_subscriber.LabelSubscriber",
            "text": "Select the objects that contain the filtered objects",
            "value": "None"
          },
          {
            "name": "cellprofiler_core.setting.text._directory.Directory",
            "text": "Select the location of the rules or classifier file",
            "value": "Elsewhere...|"
          },
          {
            "name": "cellprofiler_core.setting.text._filename.Filename",
```

"text": "Rules or classifier file name",
"value": "rules.txt"
},
{
"name": "cellprofiler_core.setting.choice._choice.Choice",
"text": "Class number",
"value": "1"
},
{
"name": "cellprofiler_core.setting._hidden_count.HiddenCount",
"text": "Measurement count",
"value": "1"
},
{
"name": "cellprofiler_core.setting._hidden_count.HiddenCount",
"text": "Additional object count",
"value": "0"
},
{
"name": "cellprofiler_core.setting.choice._choice.Choice",
"text": "Assign overlapping child to",
"value": "Both parents"
},
{
"name": "cellprofiler_core.setting._binary.Binary",
"text": "Keep removed objects as a separate set?",
"value": "No"
},
{
"name": "cellprofiler_core.setting.text.alphanumeric.name._label_name.LabelName",
"text": "Name the objects removed by the filter",
"value": "RemovedObjects"
},
{
"name": "cellprofiler_core.setting._measurement.Measurement",
"text": "Select the measurement to filter by",
"value": "AreaShape_FormFactor"
},
{
"name": "cellprofiler_core.setting._binary.Binary",
"text": "Filter using a minimum measurement value?",
"value": "Yes"
},
{
"name": "cellprofiler_core.setting.text.number._float.Float",
"text": "Minimum value",
"value": "0.5"
},
{
"name": "cellprofiler_core.setting._binary.Binary",
"text": "Filter using a maximum measurement value?",
"value": "Yes"
},
{
"name": "cellprofiler_core.setting.text.number._float.Float",
"text": "Maximum value",
"value": "1.0"

},
{
"name": "cellprofiler_core.setting._binary.Binary",
"text": "Allow fuzzy feature matching?",
"value": "No"
}
]
},
{
"attributes": {
"module_num": 10,
"notes": [],
"show_window": true,
"wants_pause": false,
"svn_version": "Unknown",
"enabled": true,
"variable_revision_number": 3,
"batch_state": "array([], dtype=uint8)",
"module_name": "MeasureObjectSizeShape",
"module_path": "cellprofiler.modules.measureobjectsizeshape.MeasureObjectSizeShape"
},
"settings": [
{
"name":
"cellprofiler_core.setting.subscriber.list_subscriber._label_list_subscriber.LabelListSubscriber",
"text": "Select object sets to measure",
"value": "FilterObjects"
},
{
"name": "cellprofiler_core.setting._binary.Binary",
"text": "Calculate the Zernike features?",
"value": "Yes"
},
{
"name": "cellprofiler_core.setting._binary.Binary",
"text": "Calculate the advanced features?",
"value": "Yes"
}
]
},
{
"attributes": {
"module_num": 11,
"notes": [],
"show_window": true,
"wants_pause": false,
"svn_version": "Unknown",
"enabled": true,
"variable_revision_number": 5,
"batch_state": "array([], dtype=uint8)",
"module_name": "MeasureImageAreaOccupied",
"module_path": "cellprofiler.modules.measureimageareaoccupied.MeasureImageAreaOccupied"
},
"settings": [
{
"name": "cellprofiler_core.setting.choice._choice.Choice",
"text": "Measure the area occupied by",

"value": "Objects"
},
{
"name":
"cellprofiler_core.setting.subscriber.list_subscriber._image_list_subscriber.ImageListSubscriber",
"text": "Select binary images to measure",
"value": "DNA"
},
{
"name":
"cellprofiler_core.setting.subscriber.list_subscriber._label_list_subscriber.LabelListSubscriber",
"text": "Select object sets to measure",
"value": "FilterObjects"
}
]
},
{
"attributes": {
"module_num": 12,
"notes": [],
"show_window": true,
"wants_pause": true,
"svn_version": "Unknown",
"enabled": true,
"variable_revision_number": 4,
"batch_state": "array([], dtype=uint8)",
"module_name": "MeasureImageIntensity",
"module_path": "cellprofiler.modules.measureimageintensity.MeasureImageIntensity"
},
"settings": [
{
"name":
"cellprofiler_core.setting.subscriber.list_subscriber._image_list_subscriber.ImageListSubscriber",
"text": "Select images to measure",
"value": "DNA"
},
{
"name": "cellprofiler_core.setting._binary.Binary",
"text": "Measure the intensity only from areas enclosed by objects?",
"value": "Yes"
},
{
"name":
"cellprofiler_core.setting.subscriber.list_subscriber._label_list_subscriber.LabelListSubscriber",
"text": "Select input object sets",
"value": "FilterObjects"
},
{
"name": "cellprofiler_core.setting._binary.Binary",
"text": "Calculate custom percentiles",
"value": "No"
},
{
"name": "cellprofiler_core.setting.text._text.Text",
"text": "Specify percentiles to measure",
"value": "10,90"
}

```
    ]
  },
  {
    "attributes": {
      "module_num": 13,
      "notes": [],
      "show_window": true,
      "wants_pause": false,
      "svn_version": "Unknown",
      "enabled": true,
      "variable_revision_number": 13,
      "batch_state": "array([], dtype=uint8)",
      "module_name": "ExportToSpreadsheet",
      "module_path": "cellprofiler.modules.exporttospreadsheet.ExportToSpreadsheet"
    },
    "settings": [
      {
        "name": "cellprofiler_core.setting.choice._custom_choice.CustomChoice",
        "text": "Select the column delimiter",
        "value": "Comma (\",\")"
      },
      {
        "name": "cellprofiler_core.setting._binary.Binary",
        "text": "Add image metadata columns to your object data file?",
        "value": "No"
      },
      {
        "name": "cellprofiler_core.setting._binary.Binary",
        "text": "Add image file and folder names to your object data file?",
        "value": "Yes"
      },
      {
        "name": "cellprofiler_core.setting._binary.Binary",
        "text": "Select the measurements to export",
        "value": "Yes"
      },
      {
        "name": "cellprofiler_core.setting._binary.Binary",
        "text": "Calculate the per-image mean values for object measurements?",
        "value": "Yes"
      },
      {
        "name": "cellprofiler_core.setting._binary.Binary",
        "text": "Calculate the per-image median values for object measurements?",
        "value": "Yes"
      },
      {
        "name": "cellprofiler_core.setting._binary.Binary",
        "text": "Calculate the per-image standard deviation values for object measurements?",
        "value": "Yes"
      },
      {
        "name": "cellprofiler_core.setting.text._directory.Directory",
        "text": "Output file location",
        "value": "Default Input Folder sub-folder|Desktop\\\\Veikka\\\\katariina_nurmi"
      },
      {
```

"name": "cellprofiler_core.setting._binary.Binary",
"text": "Create a GenePattern GCT file?",
"value": "No"
},
{
"name": "cellprofiler_core.setting.choice._choice.Choice",
"text": "Select source of sample row name",
"value": "Metadata"
},
{
"name":
"cellprofiler_core.setting.subscriber.image_subscriber._image_subscriber.ImageSubscriber",
"text": "Select the image to use as the identifier",
"value": "None"
},
{
"name": "cellprofiler_core.setting._measurement.Measurement",
"text": "Select the metadata to use as the identifier",
"value": "None"
},
{
"name": "cellprofiler_core.setting._binary.Binary",
"text": "Export all measurement types?",
"value": "Yes"
},
{
"name":
"cellprofiler_core.setting.multichoice._measurement_multichoice.MeasurementMultiChoice",
"text": "Press button to select measurements",
"value":
"Image|ModuleError_09FilterObjects,Image|ModuleError_03NamesAndTypes,Image|ModuleError_02Metadata,Image|ModuleError_08MeasureObjectSizeShape,Image|ModuleError_01Images,Image|ModuleError_04Groups,Image|ModuleError_07IdentifyPrimaryObjects,Image|ModuleError_05CorrectIlluminationCalculate,Image|ModuleError_06CorrectIlluminationApply,Image|AreaOccupied_TotalArea_FilterObjects,Image|AreaOccupied_AreaOccupied_FilterObjects,Image|AreaOccupied_Perimeter_FilterObjects,Image|Intensity_TotalArea_DNA_FilterObjects,Image|Intensity_MeanIntensity_DNA_FilterObjects,Image|Intensity_TotalIntensity_DNA_FilterObjects,Image|Intensity_PercentMaximal_DNA_FilterObjects,Image|Intensity_StdIntensity_DNA_FilterObjects,Image|Intensity_MADIntensity_DNA_FilterObjects,Image|Intensity_MaxIntensity_DNA_FilterObjects,Image|Intensity_LowerQuartileIntensity_DNA_FilterObjects,Image|Intensity_UpperQuartileIntensity_DNA_FilterObjects,Image|Intensity_MinIntensity_DNA_FilterObjects,Image|Intensity_MedianIntensity_DNA_FilterObjects,Image|Threshold_FinalThreshold_IdentifyPrimaryObjects,Image|Threshold_SumOfEntropies_IdentifyPrimaryObjects,Image|Threshold_OrigThreshold_IdentifyPrimaryObjects,Image|Threshold_WeightedVariance_IdentifyPrimaryObjects,Image|ExecutionTime_02Metadata,Image|ExecutionTime_01Images,Image|ExecutionTime_06CorrectIlluminationApply,Image|ExecutionTime_04Groups,Image|ExecutionTime_03NamesAndTypes,Image|ExecutionTime_08MeasureObjectSizeShape,Image|ExecutionTime_09FilterObjects,Image|ExecutionTime_05CorrectIlluminationCalculate,Image|ExecutionTime_07IdentifyPrimaryObjects,Image|Count_IdentifyPrimaryObjects,Image|Count_FilterObjects,Image|MD5Digest_DNA,Image|Width_DNA,Image|Height_DNA,Image|Frame_DNA,Image|PathName_DNA,Image|Group_Length,Image|Group_Number,Image|Group_Index,Image|Scaling_DNA,Image|URL_DNA,Image|FileName_DNA,Image|Series_DNA,FilterObjects|AreaShape_SpatialMoment_1_2,FilterObjects|AreaShape_SpatialMoment_1_3,FilterObjects|AreaShape_SpatialMoment_1_1,FilterObjects|AreaShape_SpatialMoment_1_0,FilterObjects|AreaShape_SpatialMoment_0_3,FilterObjects|AreaShape_SpatialMoment_0_2,FilterObjects|AreaShape_SpatialMoment_0_0,FilterObjects|AreaShape_SpatialMoment_0_1,FilterObjects|AreaShape_SpatialMoment_2_3,FilterObjects|AreaShape_SpatialMoment_2_0,FilterObjects|AreaShape_SpatialMoment_2_2,FilterObjects|AreaShape_SpatialMoment_2_1,FilterObjects|AreaShape_CentralMoment_2_0,FilterObjects|AreaShape_CentralMoment_2_2,FilterObjects|AreaShape_CentralMoment_2_1,FilterObjects|AreaShape_CentralMoment_2_3,FilterObjects|AreaShape_CentralMoment_1_2,Filt

erObjects|AreaShape_CentralMoment_1_0,FilterObjects|AreaShape_CentralMoment_1_1,FilterObjects|AreaShape_CentralMoment_1_3,FilterObjects|AreaShape_CentralMoment_0_3,FilterObjects|AreaShape_CentralMoment_0_1,FilterObjects|AreaShape_CentralMoment_0_0,FilterObjects|AreaShape_CentralMoment_0_2,FilterObjects|AreaShape_MaxFeretDiameter,FilterObjects|AreaShape_Zernike_8_4,FilterObjects|AreaShape_Zernike_8_2,FilterObjects|AreaShape_Zernike_8_8,FilterObjects|AreaShape_Zernike_8_6,FilterObjects|AreaShape_Zernike_8_0,FilterObjects|AreaShape_Zernike_7_3,FilterObjects|AreaShape_Zernike_7_1,FilterObjects|AreaShape_Zernike_7_7,FilterObjects|AreaShape_Zernike_7_5,FilterObjects|AreaShape_Zernike_1_1,FilterObjects|AreaShape_Zernike_6_6,FilterObjects|AreaShape_Zernike_6_2,FilterObjects|AreaShape_Zernike_6_0,FilterObjects|AreaShape_Zernike_6_4,FilterObjects|AreaShape_Zernike_2_0,FilterObjects|AreaShape_Zernike_2_2,FilterObjects|AreaShape_Zernike_3_3,FilterObjects|AreaShape_Zernike_3_1,FilterObjects|AreaShape_Zernike_4_2,FilterObjects|AreaShape_Zernike_4_0,FilterObjects|AreaShape_Zernike_4_4,FilterObjects|AreaShape_Zernike_9_9,FilterObjects|AreaShape_Zernike_9_5,FilterObjects|AreaShape_Zernike_9_7,FilterObjects|AreaShape_Zernike_9_1,FilterObjects|AreaShape_Zernike_9_3,FilterObjects|AreaShape_Zernike_0_0,FilterObjects|AreaShape_Zernike_5_3,FilterObjects|AreaShape_Zernike_5_5,FilterObjects|AreaShape_Zernike_5_1,FilterObjects|AreaShape_ConvexArea,FilterObjects|AreaShape_NormalizedMoment_0_3,FilterObjects|AreaShape_NormalizedMoment_0_0,FilterObjects|AreaShape_NormalizedMoment_0_2,FilterObjects|AreaShape_NormalizedMoment_0_1,FilterObjects|AreaShape_NormalizedMoment_3_2,FilterObjects|AreaShape_NormalizedMoment_3_0,FilterObjects|AreaShape_NormalizedMoment_3_3,FilterObjects|AreaShape_NormalizedMoment_3_1,FilterObjects|AreaShape_NormalizedMoment_1_0,FilterObjects|AreaShape_NormalizedMoment_1_2,FilterObjects|AreaShape_NormalizedMoment_1_3,FilterObjects|AreaShape_NormalizedMoment_1_1,FilterObjects|AreaShape_NormalizedMoment_2_1,FilterObjects|AreaShape_NormalizedMoment_2_2,FilterObjects|AreaShape_NormalizedMoment_2_0,FilterObjects|AreaShape_NormalizedMoment_2_3,FilterObjects|AreaShape_HuMoment_5,FilterObjects|AreaShape_HuMoment_4,FilterObjects|AreaShape_HuMoment_2,FilterObjects|AreaShape_HuMoment_6,FilterObjects|AreaShape_HuMoment_0,FilterObjects|AreaShape_HuMoment_1,FilterObjects|AreaShape_HuMoment_3,FilterObjects|AreaShape_Solidity,FilterObjects|AreaShape_InertiaTensor_1_0,FilterObjects|AreaShape_InertiaTensor_1_1,FilterObjects|AreaShape_InertiaTensor_0_1,FilterObjects|AreaShape_InertiaTensor_0_0,FilterObjects|AreaShape_Compactness,FilterObjects|AreaShape_Extent,FilterObjects|AreaShape_MaximumRadius,FilterObjects|AreaShape_BoundingBoxMaximum_X,FilterObjects|AreaShape_BoundingBoxMaximum_Y,FilterObjects|AreaShape_Orientation,FilterObjects|AreaShape_FormFactor,FilterObjects|AreaShape_MeanRadius,FilterObjects|AreaShape_InertiaTensorEigenvalues_0,FilterObjects|AreaShape_InertiaTensorEigenvalues_1,FilterObjects|AreaShape_BoundingBoxMinimum_X,FilterObjects|AreaShape_BoundingBoxMinimum_Y,FilterObjects|AreaShape_Center_X,FilterObjects|AreaShape_Center_Y,FilterObjects|AreaShape_MinFeretDiameter,FilterObjects|AreaShape_Eccentricity,FilterObjects|AreaShape_MedianRadius,FilterObjects|AreaShape_EquivalentDiameter,FilterObjects|AreaShape_MinorAxisLength,FilterObjects|AreaShape_Area,FilterObjects|AreaShape_EulerNumber,FilterObjects|AreaShape_BoundingBoxArea,FilterObjects|AreaShape_Perimeter,FilterObjects|AreaShape_MajorAxisLength,FilterObjects|Location_Center_X,FilterObjects|Location_Center_Y,FilterObjects|Location_Center_Z,FilterObjects|Number_Object_Number,FilterObjects|Parent_IdentifyPrimaryObjects,Experiment|Run_Timestamp,Experiment|Modification_Timestamp,Experiment|Pipeline_Pipeline,Experiment|CellProfiler_Version"
},
{
"name": "cellprofiler_core.setting.choice._choice.Choice",
"text": "Representation of Nan/Inf",
"value": "NaN"
},
{
"name": "cellprofiler_core.setting._binary.Binary",
"text": "Add a prefix to file names?",
"value": "Yes"
},
{
"name": "cellprofiler_core.setting.text._text.Text",
"text": "Filename prefix",
"value": "Filtered_AD_VCAM"
},
{

```
            "name": "cellprofiler_core.setting._binary.Binary",
            "text": "Overwrite existing files without warning?",
            "value": "No"
        },
        {
            "name": "cellprofiler.modules.exporttospreadsheet.EEObjectNameSubscriber",
            "text": "Data to export",
            "value": "Do not use"
        },
        {
            "name": "cellprofiler_core.setting._binary.Binary",
            "text": "Combine these object measurements with those of the previous object?",
            "value": "No"
        },
        {
            "name": "cellprofiler_core.setting.text._text.Text",
            "text": "File name",
            "value": "DATA.csv"
        },
        {
            "name": "cellprofiler_core.setting._binary.Binary",
            "text": "Use the object name for the file name?",
            "value": "Yes"
        }
    ]
},
{
    "attributes": {
        "module_num": 14,
        "notes": [],
        "show_window": true,
        "wants_pause": false,
        "svn_version": "Unknown",
        "enabled": true,
        "variable_revision_number": 13,
        "batch_state": "array([], dtype=uint8)",
        "module_name": "ExportToSpreadsheet",
        "module_path": "cellprofiler.modules.exporttospreadsheet.ExportToSpreadsheet"
    },
    "settings": [
        {
            "name": "cellprofiler_core.setting.choice._custom_choice.CustomChoice",
            "text": "Select the column delimiter",
            "value": "Comma (\",\")"
        },
        {
            "name": "cellprofiler_core.setting._binary.Binary",
            "text": "Add image metadata columns to your object data file?",
            "value": "No"
        },
        {
            "name": "cellprofiler_core.setting._binary.Binary",
            "text": "Add image file and folder names to your object data file?",
            "value": "No"
        },
        {
            "name": "cellprofiler_core.setting._binary.Binary",
```

"text": "Select the measurements to export",
"value": "No"
},
{
"name": "cellprofiler_core.setting._binary.Binary",
"text": "Calculate the per-image mean values for object measurements?",
"value": "No"
},
{
"name": "cellprofiler_core.setting._binary.Binary",
"text": "Calculate the per-image median values for object measurements?",
"value": "No"
},
{
"name": "cellprofiler_core.setting._binary.Binary",
"text": "Calculate the per-image standard deviation values for object measurements?",
"value": "No"
},
{
"name": "cellprofiler_core.setting.text._directory.Directory",
"text": "Output file location",
"value": "Default Output Folder|"
},
{
"name": "cellprofiler_core.setting._binary.Binary",
"text": "Create a GenePattern GCT file?",
"value": "No"
},
{
"name": "cellprofiler_core.setting.choice._choice.Choice",
"text": "Select source of sample row name",
"value": "Metadata"
},
{
"name":
"cellprofiler_core.setting.subscriber.image_subscriber._image_subscriber.ImageSubscriber",
"text": "Select the image to use as the identifier",
"value": "None"
},
{
"name": "cellprofiler_core.setting._measurement.Measurement",
"text": "Select the metadata to use as the identifier",
"value": "None"
},
{
"name": "cellprofiler_core.setting._binary.Binary",
"text": "Export all measurement types?",
"value": "Yes"
},
{
"name":
"cellprofiler_core.setting.multichoice._measurement_multichoice.MeasurementMultiChoice",
"text": "Press button to select measurements",
"value": ""
},
{
"name": "cellprofiler_core.setting.choice._choice.Choice",

```
                "text": "Representation of Nan/Inf",
                "value": "NaN"
            },
            {
                "name": "cellprofiler_core.setting._binary.Binary",
                "text": "Add a prefix to file names?",
                "value": "Yes"
            },
            {
                "name": "cellprofiler_core.setting.text._text.Text",
                "text": "Filename prefix",
                "value": "MyExpt_"
            },
            {
                "name": "cellprofiler_core.setting._binary.Binary",
                "text": "Overwrite existing files without warning?",
                "value": "No"
            },
            {
                "name": "cellprofiler.modules.exporttospreadsheet.EEObjectNameSubscriber",
                "text": "Data to export",
                "value": "Do not use"
            },
            {
                "name": "cellprofiler_core.setting._binary.Binary",
                "text": "Combine these object measurements with those of the previous object?",
                "value": "No"
            },
            {
                "name": "cellprofiler_core.setting.text._text.Text",
                "text": "File name",
                "value": "DATA.csv"
            },
            {
                "name": "cellprofiler_core.setting._binary.Binary",
                "text": "Use the object name for the file name?",
                "value": "Yes"
            }
        ]
    }
  ],
  "version": "v6"
}
```

Source data

Lipopolysaccharides Drive Proinflammatory Extracellular Vesicle Secretion in Coronary Artery Endothelial Cells via Noncanonical Inflammasome Activation

Katariina Nurmi (ORCID 0000-0001-5406-5334)[1]*, Martina B. Lorey (ORCID 0000-0001-5605-6714)[2,3]*, Jukka Parantainen[1], Wojciech Cypryk[4], Eirini Kalogerakou[1], Vesa-Petteri Kouri[1], Juha Kaivola[1], Marcelina Bilicka[1], Arzu Beklen[1,5], Yan Chen[6], Maria Stensland[7], Sampsa Matikainen[1], Tuula A. Nyman[7]#, Kari K. Eklund[1,8,9]#

*Contributed equally
#Contributed equally

[1] *Faculty of Medicine, Clinicum, Translational Immunology Program, University of Helsinki, Helsinki, Finland*
[2] *Atherosclerosis Research Laboratory, Wihuri Research Institute, Helsinki, Finland.*
[3] *Faculty of Biological and Environmental Sciences, Molecular and Integrative Biosciences Research Programme, University of Helsinki, Helsinki, Finland*
[4] *Centre of Molecular and Macromolecular Studies, Polish Academy of Sciences, Lodz, Poland*
[5] *Faculty of Dentistry, Eskisehir Osmangazi University, Eskisehir, Turkey.*
[6] *Urological department, Institute of Clinical Medicine, the First Affiliated Hospital of Zhengzhou University, Zhengzhou City, Hennan Province, China.*
[7] *Department of Immunology, University of Oslo and Oslo University Hospital, Oslo, Norway*
[8] *Department of Rheumatology, University of Helsinki and Helsinki University Hospital, Helsinki, Finland*
[9] *ORTON Orthopaedic Hospital, Helsinki, Finland*

Correspondence to: Katariina.nurmi@helsinki.fi

Fig.2 Source data

a Membranes were cut at 75 kDa for blot analysis, and the lower part was blotted for IL-1β

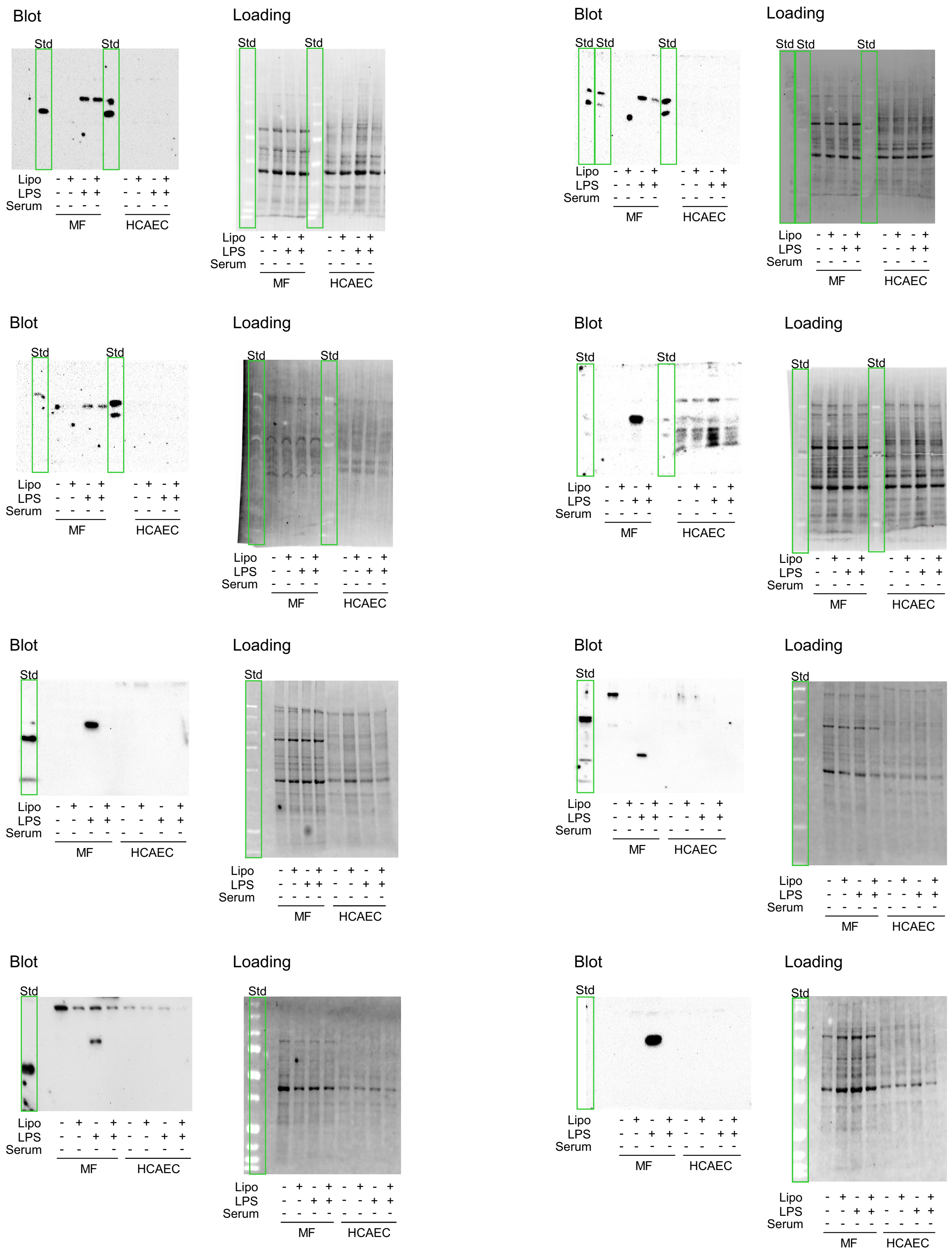

Fig.2 Source data

b Membrane was cut at 75 kDa for blot analysis, the upper part was blotted for NLRP3

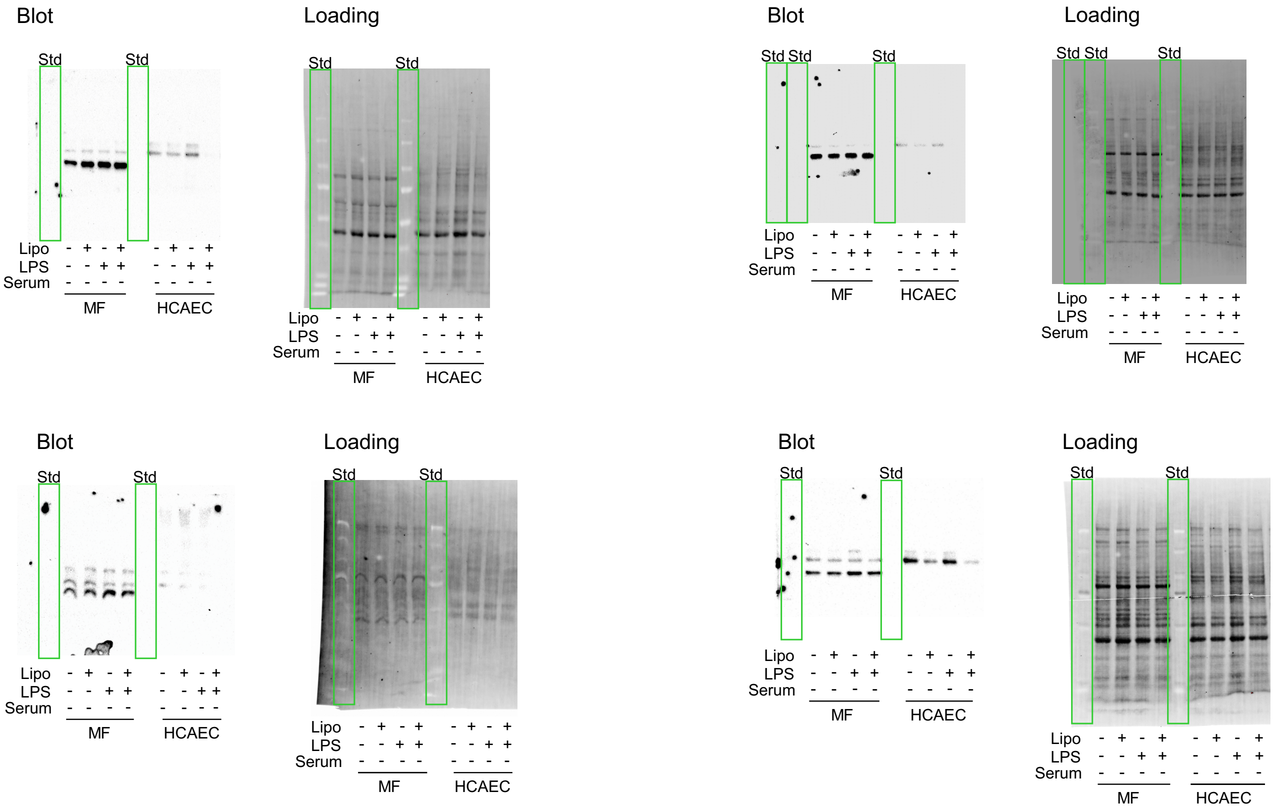

Fig.3 Source data

e

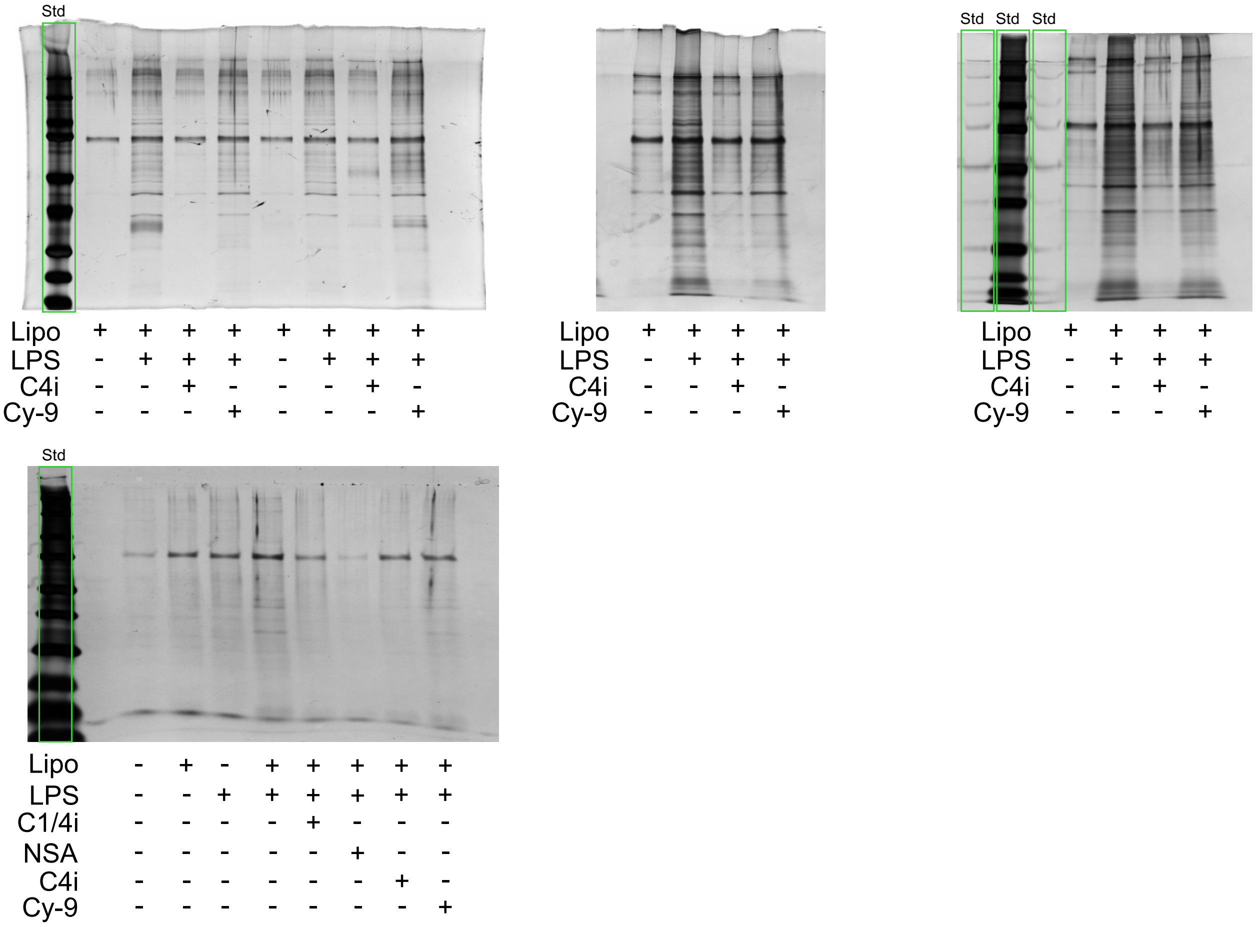

f

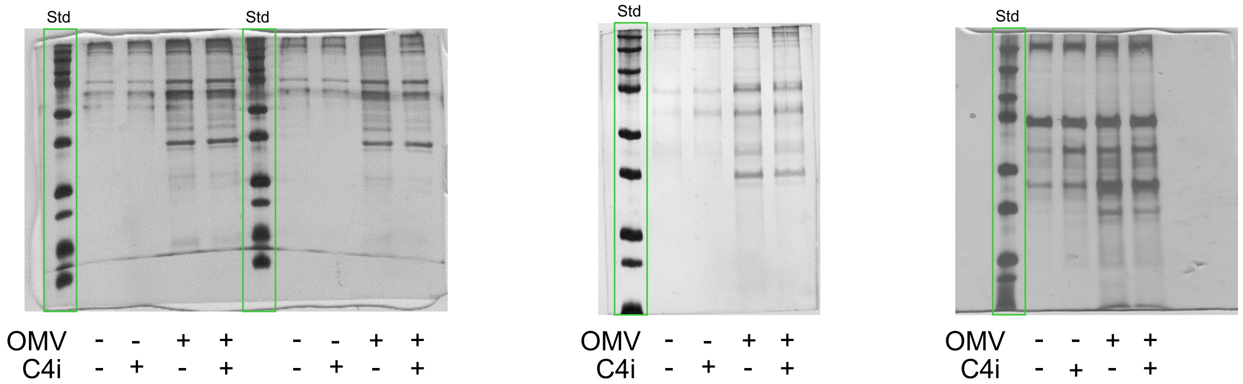

Fig.5 Source data

b

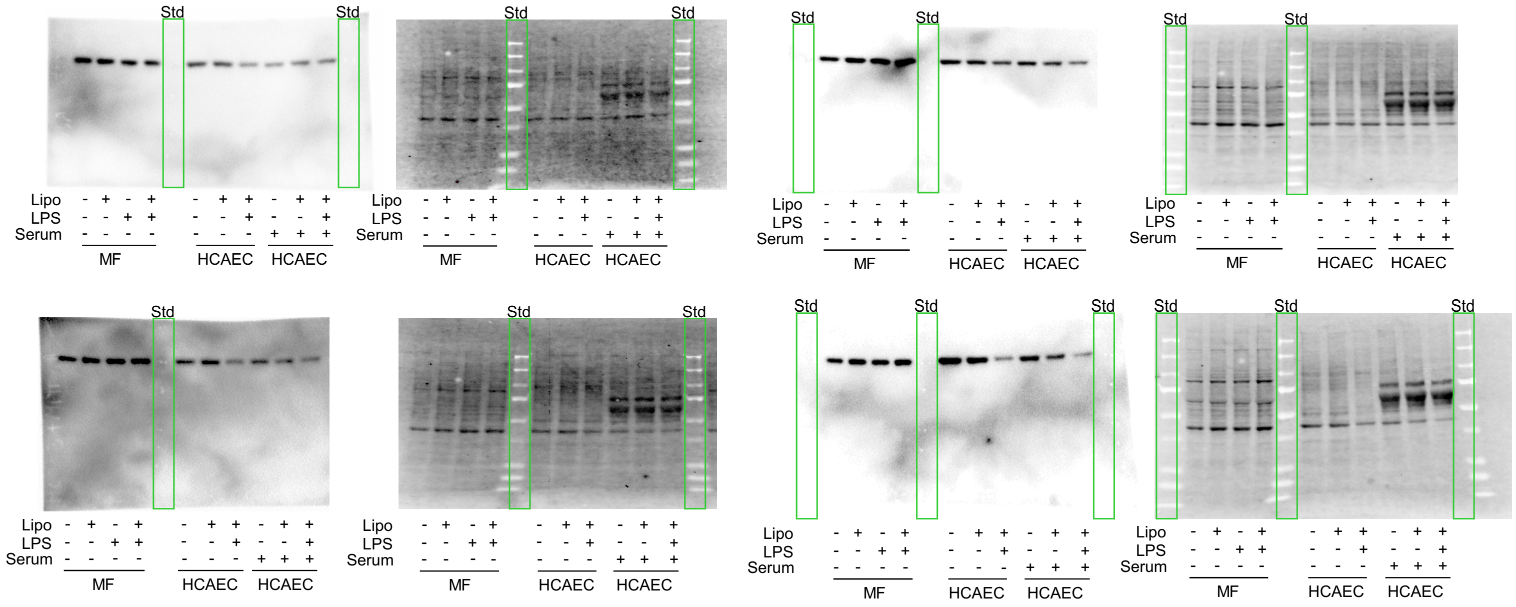

d

N-terminal GSDMD was blotted on a stripped membrane cut at 75 kDa, remaining MLKL staining is shown around 50 kDa

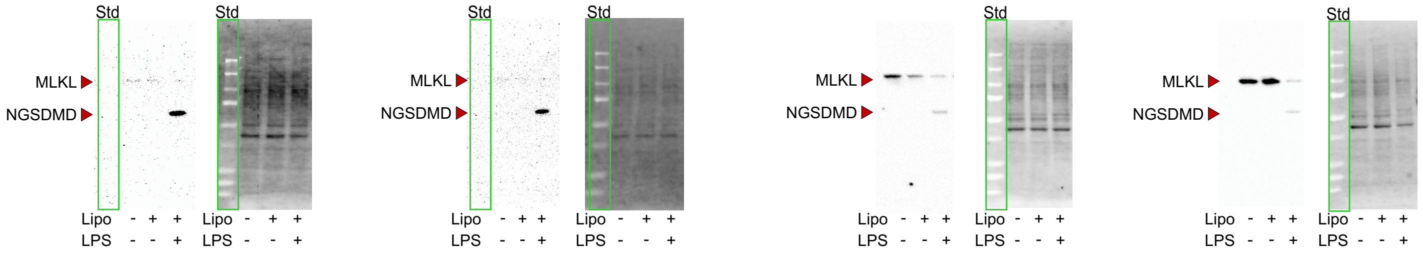

e

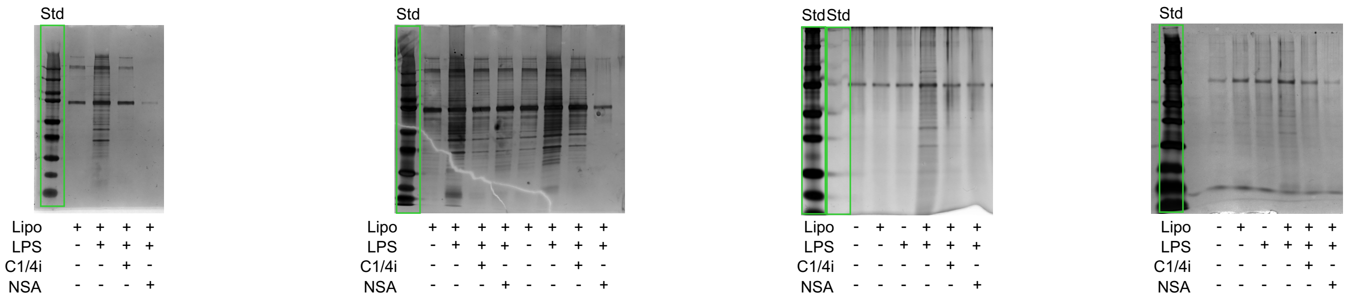

f

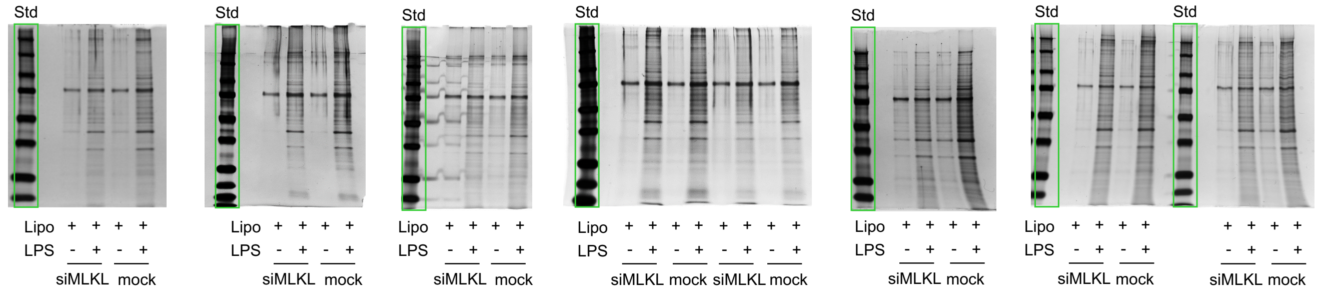

g

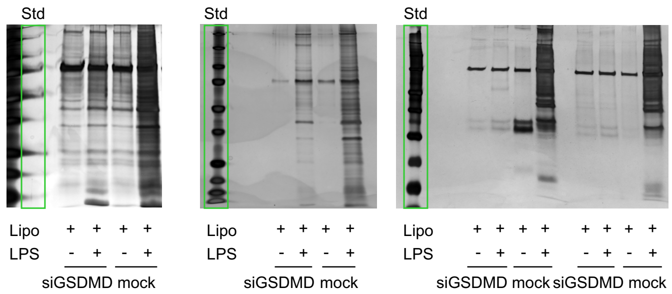

Supplementary Fig.1 Source data

c IL-1β

Membranes were cut at 75 kDa for blot analysis, and the lower part was blotted for IL-1β

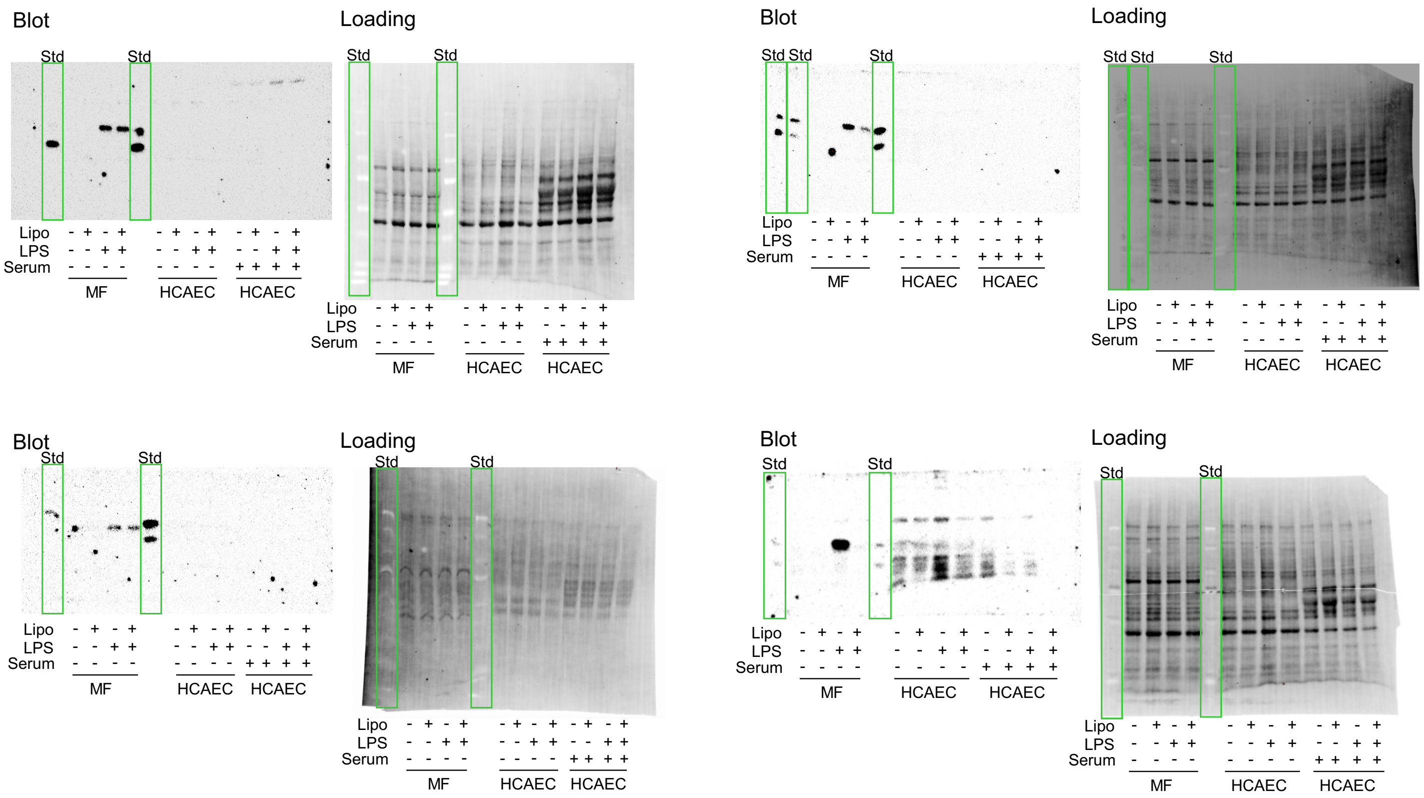

c NLRP3

Membrane was cut at 75 kDa for blot analysis, the upper part was blotted for NLRP3

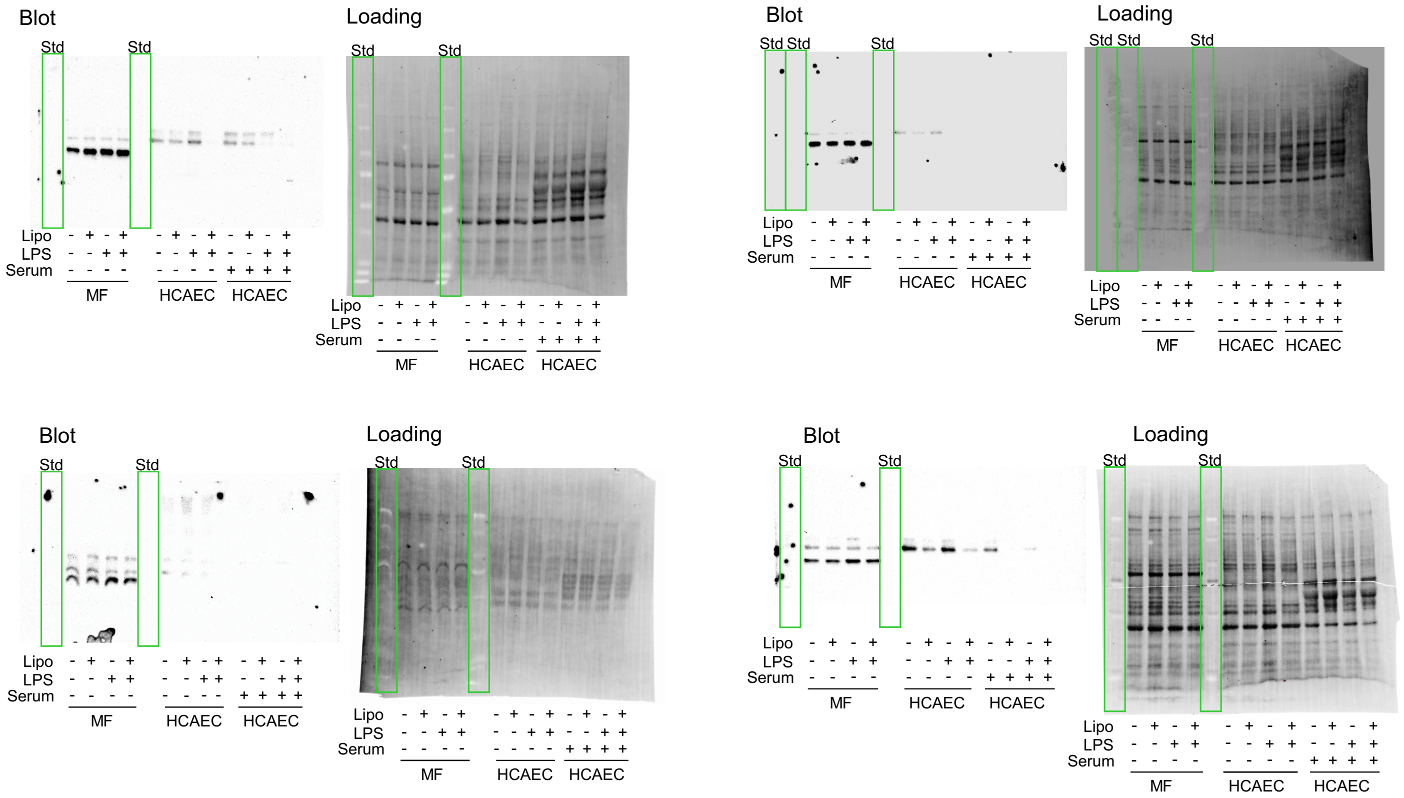

Supplementary Fig.2 Source data

a

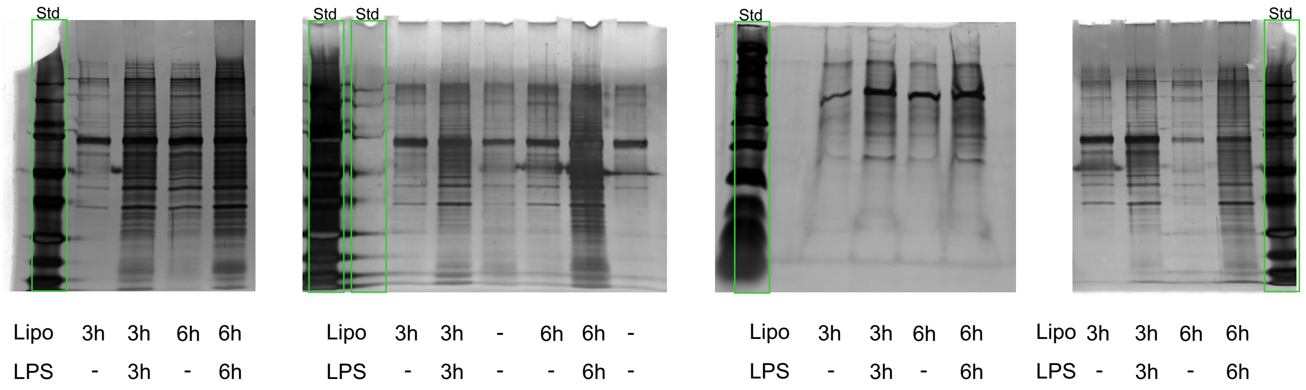

b

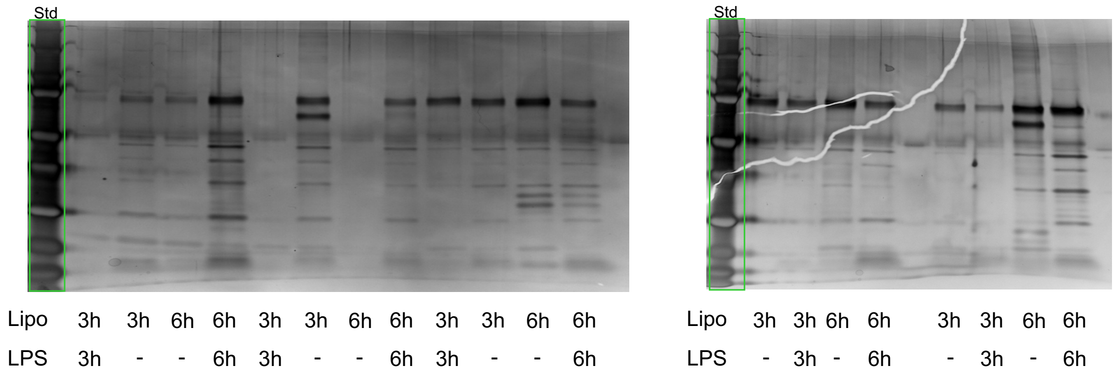

c

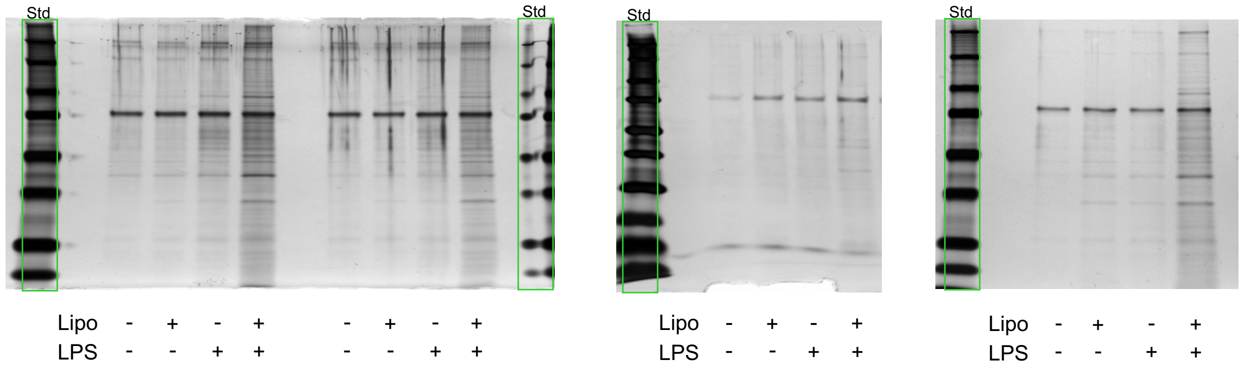

Supplementary Fig.5 Source data

c Membrane was cut at 75 kDa for blot analysis,
the lower part was blotted for MLKL

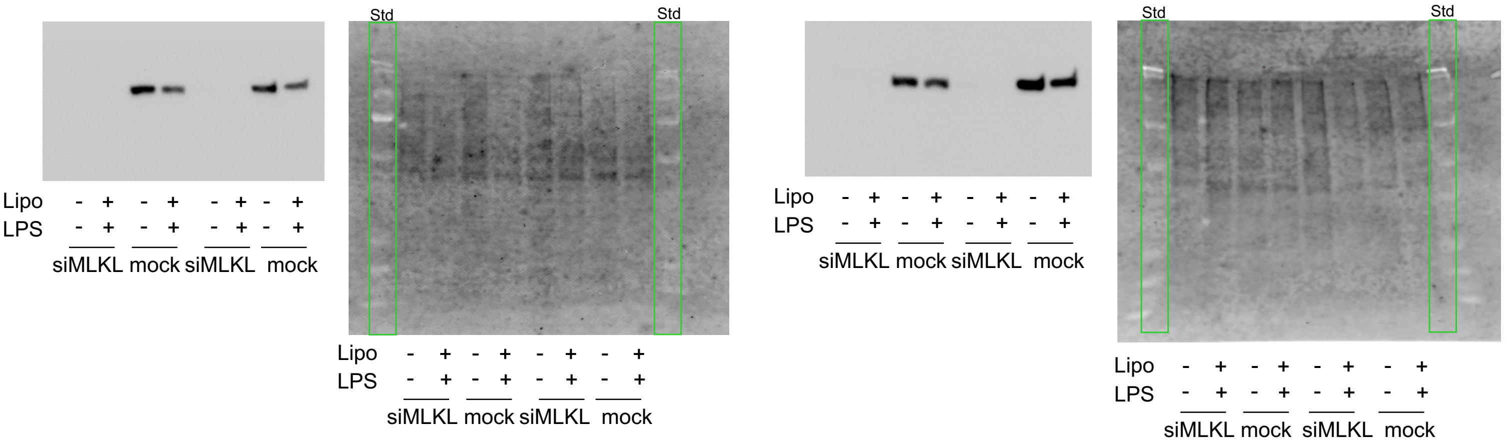

d Membrane was cut at 75 kDa for blot analysis,
the lower part was blotted for NGSDMD

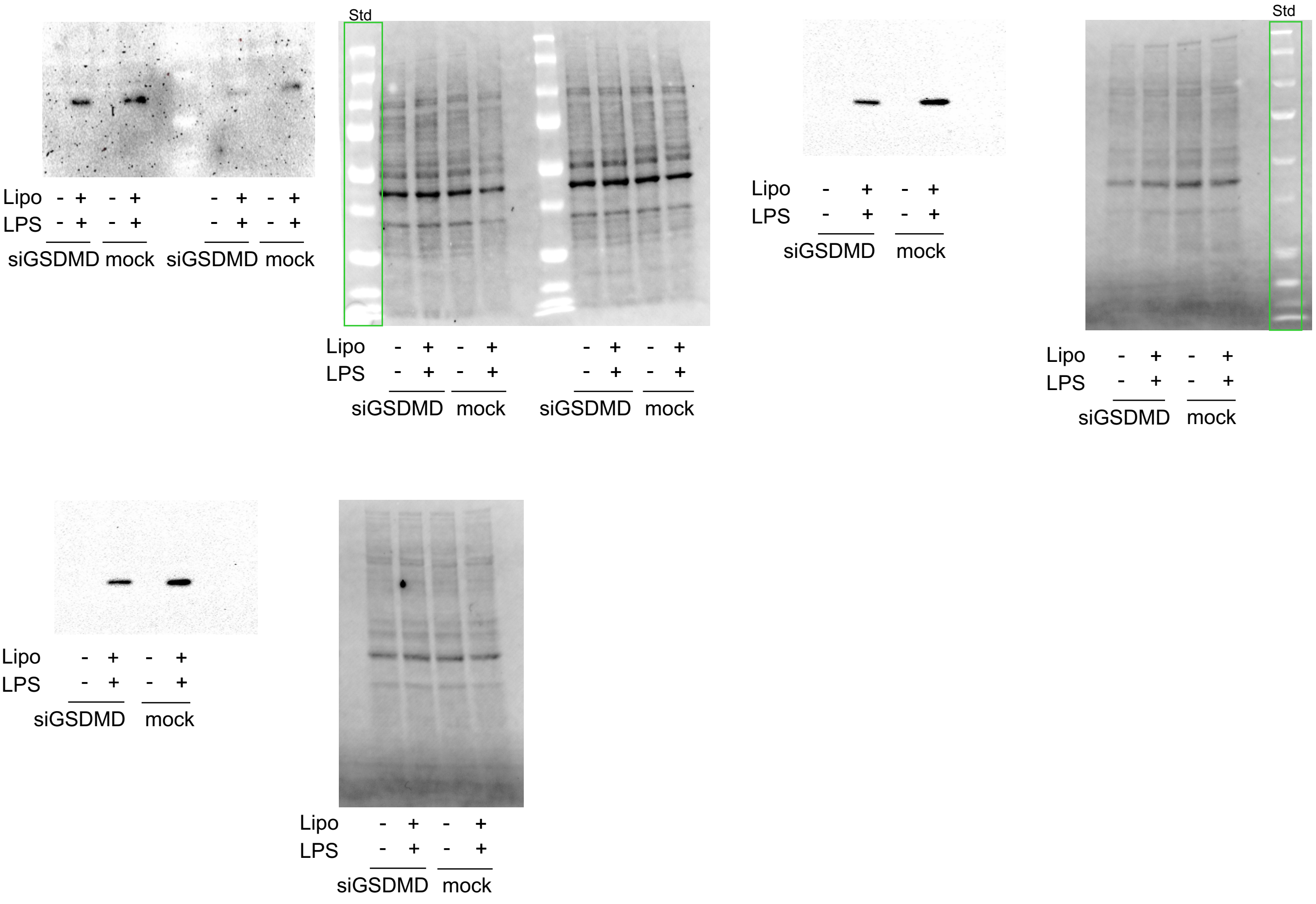

Supplement: Supplementary file 1 — Supplementary Material 1 Table S1 (PDF 27.3 MB ) [file 18_2025_6006_MOESM1_ESM.pdf]
