# Supplementary material for: Lipopolysaccharides Drive Proinflammatory Extracellular Vesicle Secretion in Coronary Artery Endothelial Cells via Noncanonical Inflammasome Activation
Source: Cell Mol Life Sci. 2026 Apr 21;83(1):239. doi: 10.1007/s00018-025-06006-y (PMC13237317; doi:10.1007/s00018-025-06006-y)
Supplement: Supplementary file 2 — Supplementary Material 2 (PDF 6.18 MB ) [file 18_2025_6006_MOESM2_ESM.pdf]

Fig.S1

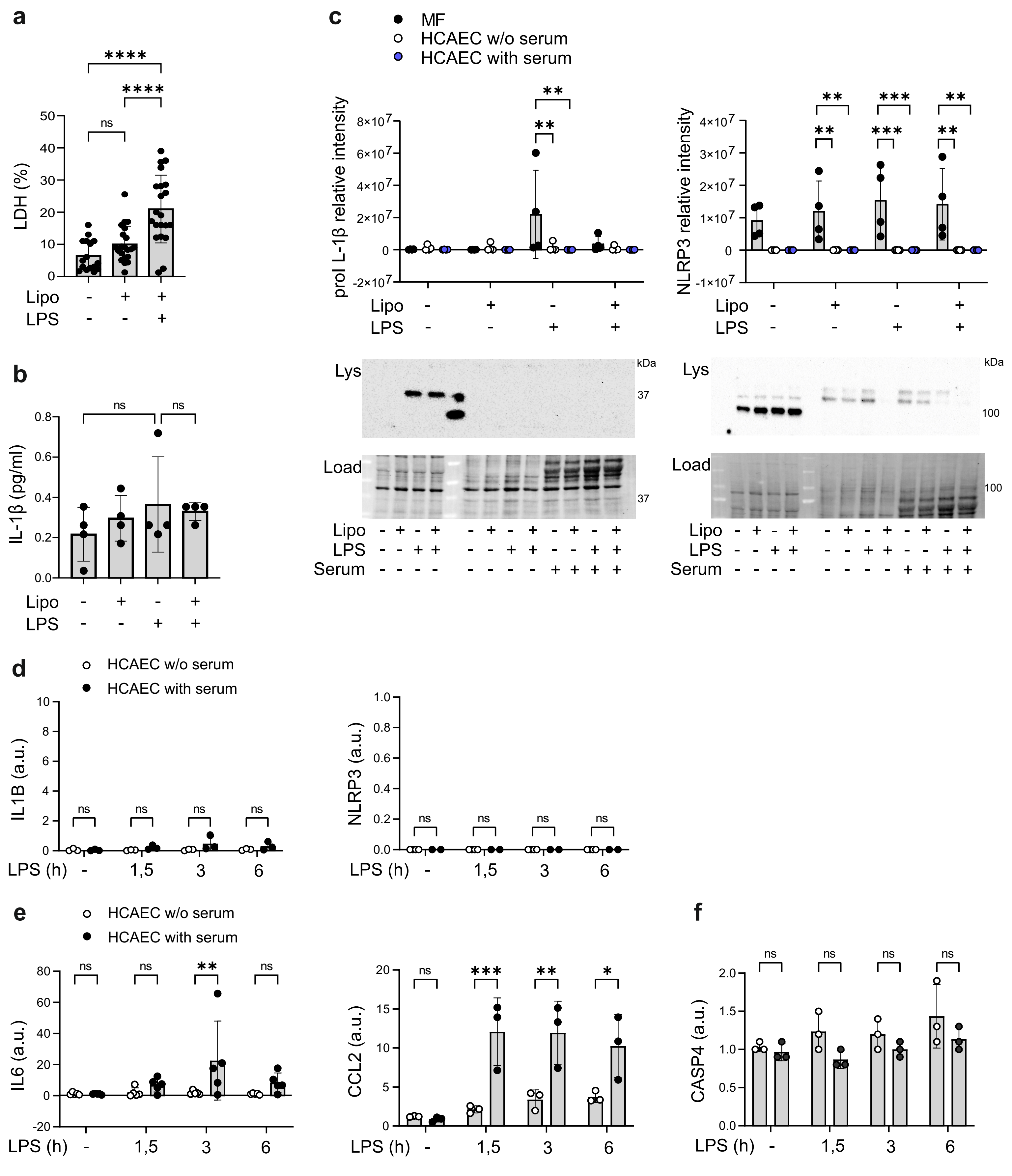

Fig. S1 NLRP3 inflammasome components are not induced in HCAECs in serum containing media
a) LDH data from panels 1a,1e, and 1g were pooled to assess the effect of lipofectamine treatment. **b)** HCAECs were transfected with a complex of ultra-pure LPS (2 µg/ml) and lipofectamine (5 µl/ml) or mock-treated with lipofectamine (5 µl/ml) or activated with extracellular LPS (1 µg/ml) for 6 h, and secretion of IL-1β was detected by ELISA. **c)** Human primary macrophages and HCAECs in serum free or complete media were transfected with a complex of ultra-pure LPS (2 µg/ml) and lipofectamine (5 µl/ml) or mock-treated with lipofectamine (5 µl/ml) or activated with extracellular LPS (1 µg/ml) for 6 h, and protein expression of proIL-1β and NLRP3 was analyzed by Western blotting from cell lysates (Lys). Representative Western blots and loading are shown. HCAECs were stimulated with extracellular LPS (1 µg/ml) for indicated times in serum free or complete media and expression of **d)** *IL1B* and *NLRP3* were analyzed by RT qPCR. **e-f)** HCAECs were stimulated with extracellular LPS (1 µg/ml) for indicated times in serum free or complete media and expression of **e)** *IL6* and *CCL2* or **f)** *CASP4* was detected by RT qPCR. Statistics: a-b, e-f) One-way or two-way ANOVA followed by Sidak´s multiple comparisons test. c, d) Two-way ANOVA followed by Tukey´s multiple comparisons test, a) n=16 – 23, b-f) n=4.

Fig.S2

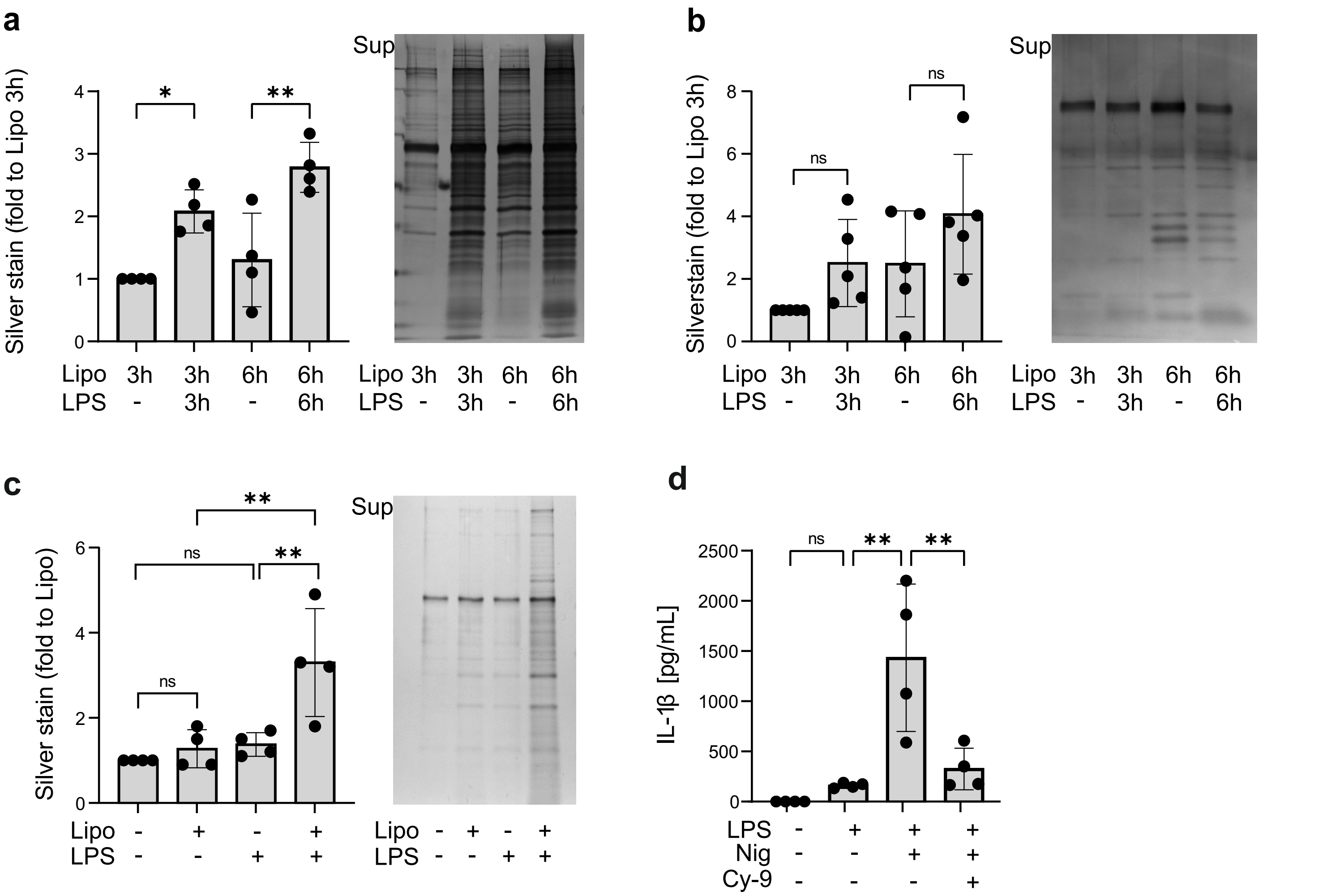

Fig. S2 Only intracellular, not extracellular, LPS affects protein secretion in extracellular vesicle fraction but does not affect conventional protein secretion
a) HCAECs were transfected with a complex of ultra-pure LPS (2 µg/ml) and lipofectamine (5 µl/ml) or mock-treated with lipofectamine (5 µl/ml) for indicated times and proteins in the EV fraction were separated by SDS-PAGE and visualized with silver staining. Representative SDS-PAGE gel shown. **b)** HCAECs were transfected with a complex of ultra-pure LPS (2 µg/ml) and lipofectamine (5 µl/ml) or mock-treated with lipofectamine (5 µl/ml) for indicated times and proteins in the rest secretome fraction (< 100 kDa proteins) were separated by SDS-PAGE and visualized with silver staining. Representative SDS-PAGE gel shown. **c)** HCAECs were stimulated with ultra-pure LPS (2 µg/ml) and/or lipofectamine (5 µl/ml) or mock-treated with lipofectamine (5 µl/ml) for 6 h. Proteins in the EV fraction were separated by SDS-PAGE and visualized with silver staining. Representative SDS-PAGE gel shown. **d)** THP-1 monocytes were activated with LPS for 4 h after which an inhibitor of NLRP3 inflammasome (Cy-9,10 µM) was added 1 h before activation with nigericin (nig; 4 µM, 1 h), and IL-1β secretion was measured by ELISA. Statistics:
a-d) One-way ANOVA followed by Sidak´s multiple comparisons test. a) n=4, b) n=5, c-d) n=4.

Fig.S3

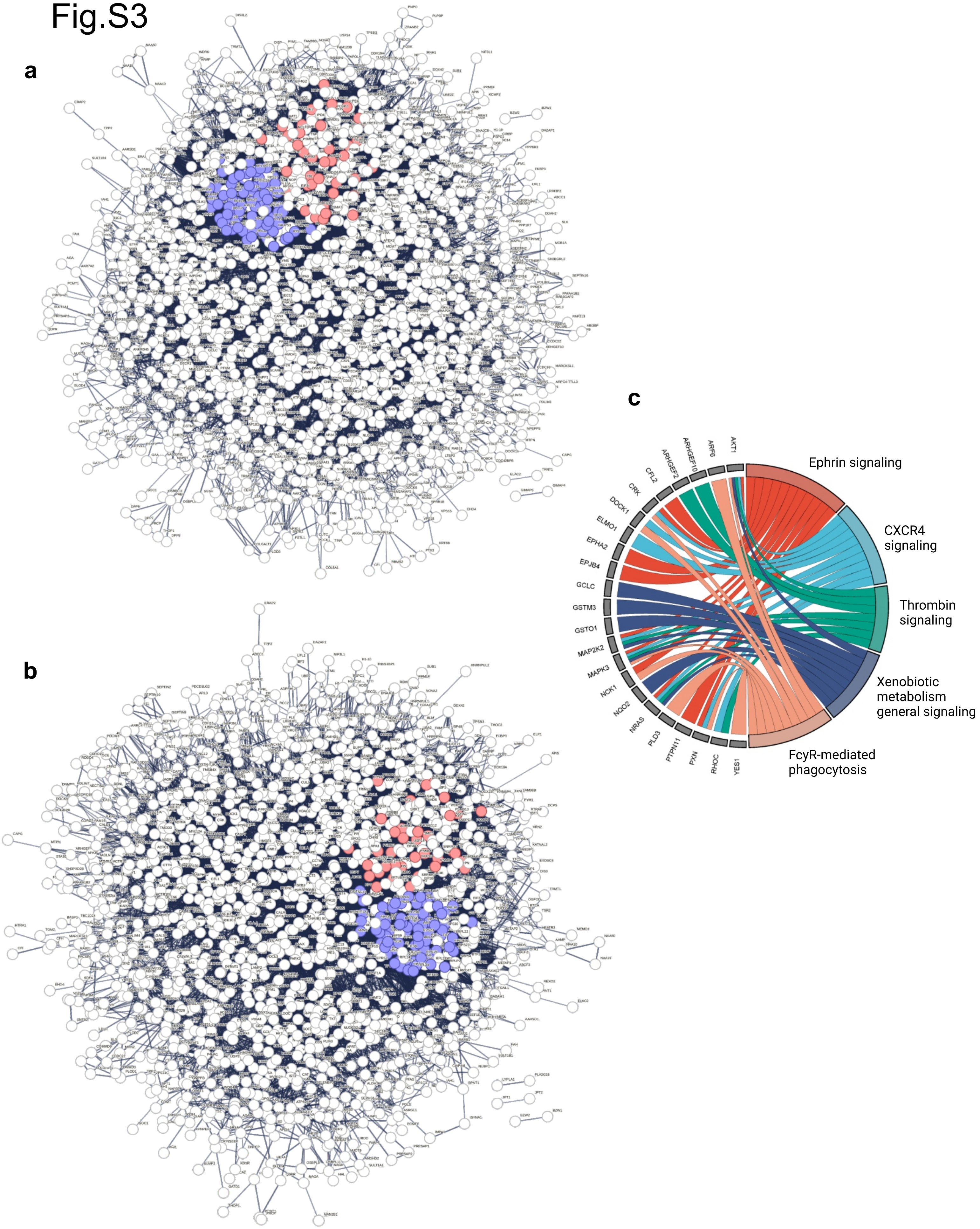

Fig. S3 STRING enrichment analyses
Protein-protein interaction network of proteins differentially enriched in EVs of LPS transfected cells at **a)** 3 h and **b)** 6 h. Interactions are visualized as lines; the line thickness indicates the strength of the interaction. Only interactions with a minimum confidence score of 0.7 (high confidence) are shown, proteins disconnected from the network are hidden. Proteins belonging to enriched KEGG pathways are colored in red when they belong to the proteasome (hsa03050 Proteasome), blue when they belong to the ribosome (hsa03010 Ribosome). **c)** Chord diagram visualizing the 5 most upregulated pathways and the proteins responsible for the enrichment in the 6 h dataset.

Fig.S4

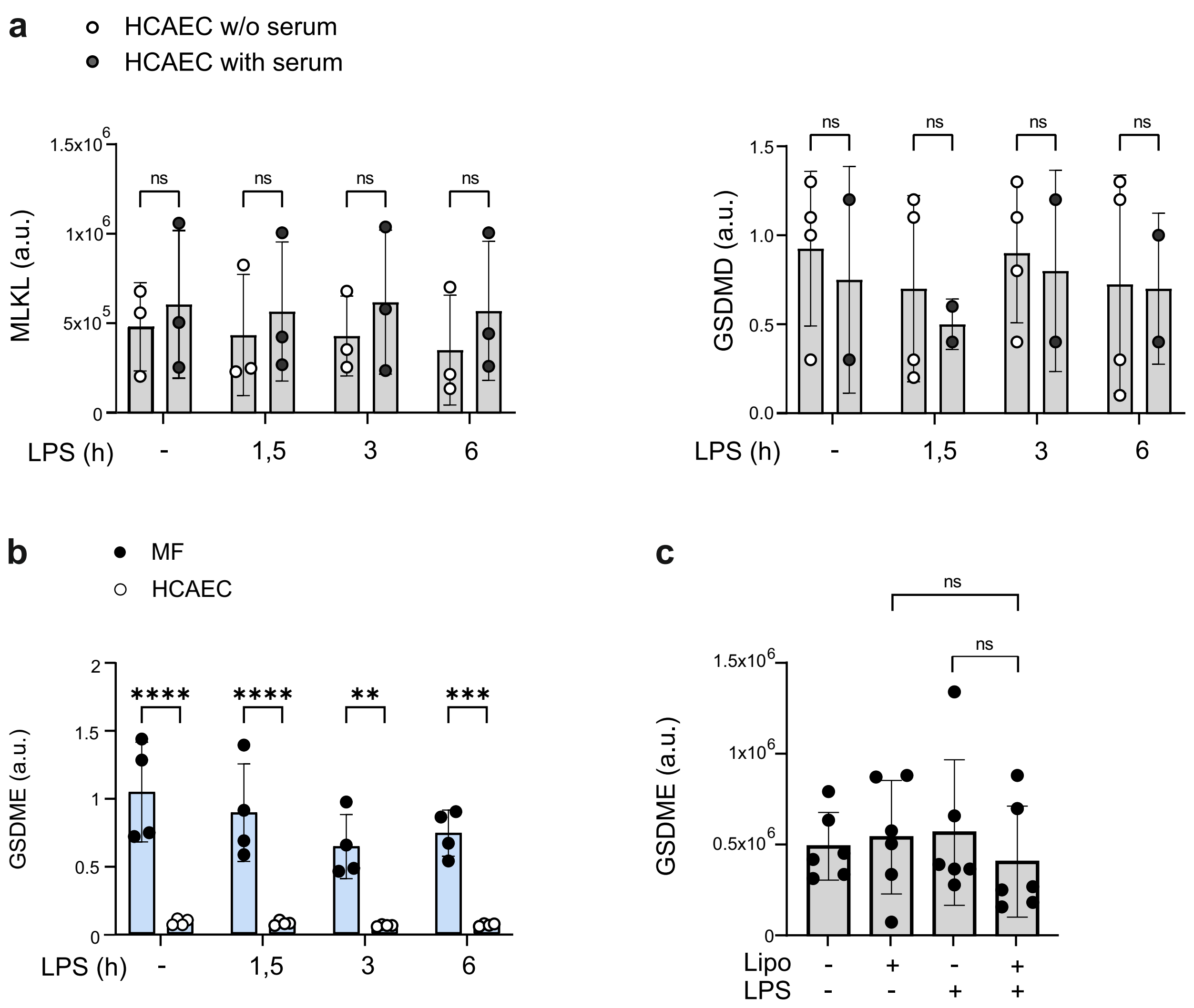

Fig. S4 Pore forming proteins are more expressed in macrophages compared to endothelial cells and in HCAECs their expression is not modified by the presence of serum
a) HCAECs were stimulated with extracellular LPS (1 µg/ml) for indicated times in serum free or complete media and expression of *MLKL* and *GSDMD* was detected by RT qPCR. **b)** HCAECs and human primary macrophages were stimulated with extracellular LPS for indicated times and mRNA expression of *GSDME* was analyzed by RT qPCR. **c)** HCAECs were transfected with a complex of ultra-pure LPS (2 µg/ml) and lipofectamine (5 µl/ml), mock-treated with lipofectamine (5 µl/ml) or stimulated with extracellular ultra-pure LPS (2 µg/ml) for 6 h and expression of *GSDME* was detected by RT qPCR. Statistics: a) Two-way ANOVA followed by Tukey´s multiple comparisons test, b) Two-way ANOVA followed by Sidak´s multiple comparisons test, c) One-way ANOVA followed by Sidak´s multiple comparisons test. a) n=3, b) n=4, c) n=6.

Fig.S5

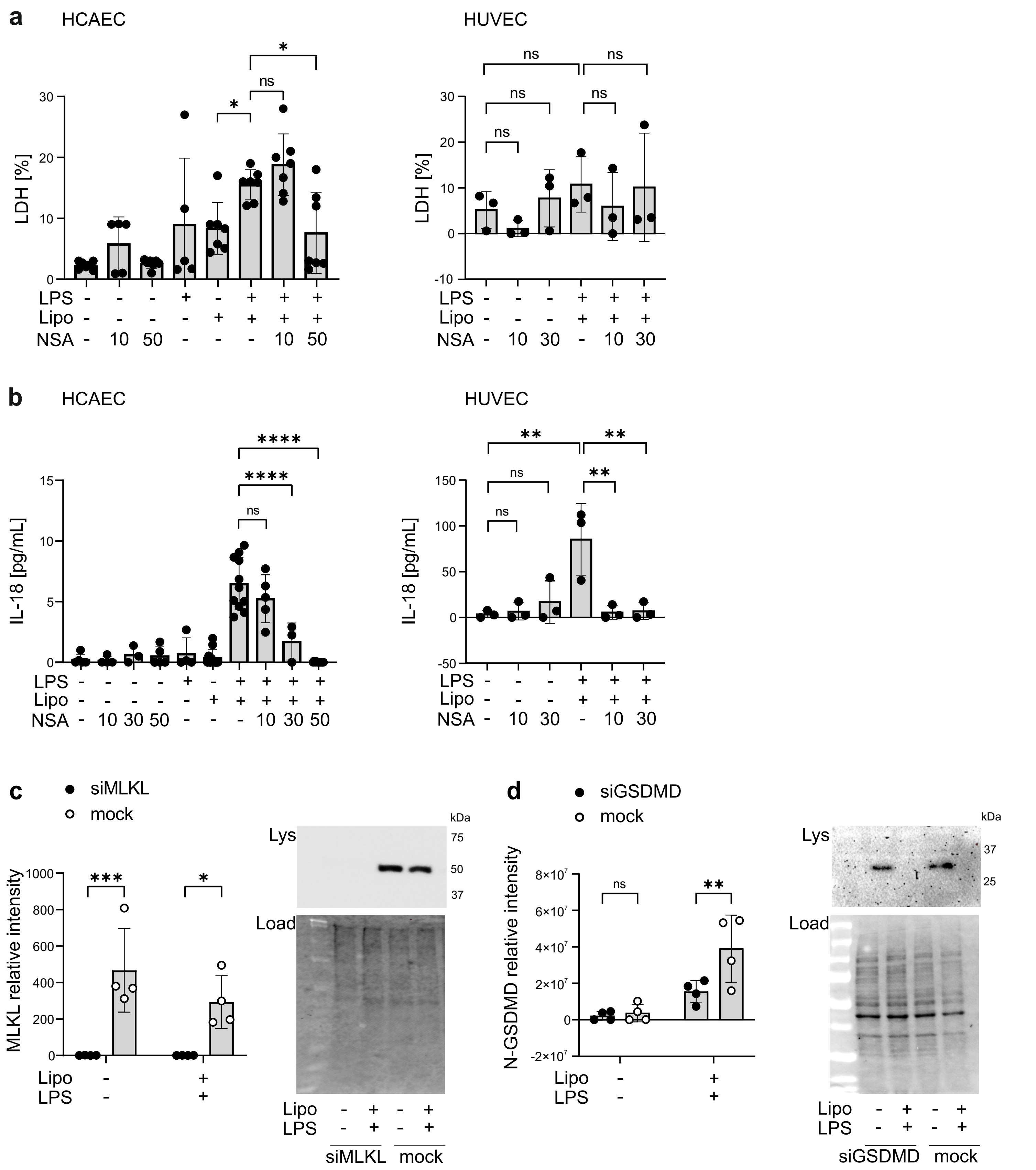

Fig. S5 Inhibition of pore forming proteins reduces pyroptotic cell death and secretion of IL-18
HCAECs and HUVECs were treated with an inhibitor of MLKL and GSDMD, necrosulfonamide (NSA, 10, 30, and 50 µM), for 1 h and then transfected with a complex of ultra-pure LPS (2 µg/ml) and lipofectamine (5 µl/ml) or mock-treated with lipofectamine (5 µl/ml) or stimulated with extracellular ultra-pure LPS (2 µg/ml) for 6 h and secretion of **a**) LDH or **b)** IL-18 was measured. HCAECs were transfected with **c)** MLKL (30 pmol/ml), **d)** GSDMD (30 pmol/ml) or mock siRNA (30 pmol/ml) using RNAiMAX (3 µl/ml), and 72 h later noncanonical inflammasome was stimulated by transfection of ultra-pure LPS (LPS 2 µg/ml with lipofectamine 5 µl/ml, 6 h). The expression of **c)** MLKL or **d)** N-GSDMD was analyzed by Western blotting from cell lysates (Lys). Representative Western blots and loading are shown.Statistics: a, b) One-way ANOVA followed by Sidak´s multiple comparisons test, c-d) Two-way ANOVA followed by Sidak´s multiple comparisons test. a, b) HCAEC n=5-11, HUVEC n=3, c-d) n=4.

Fig.S6

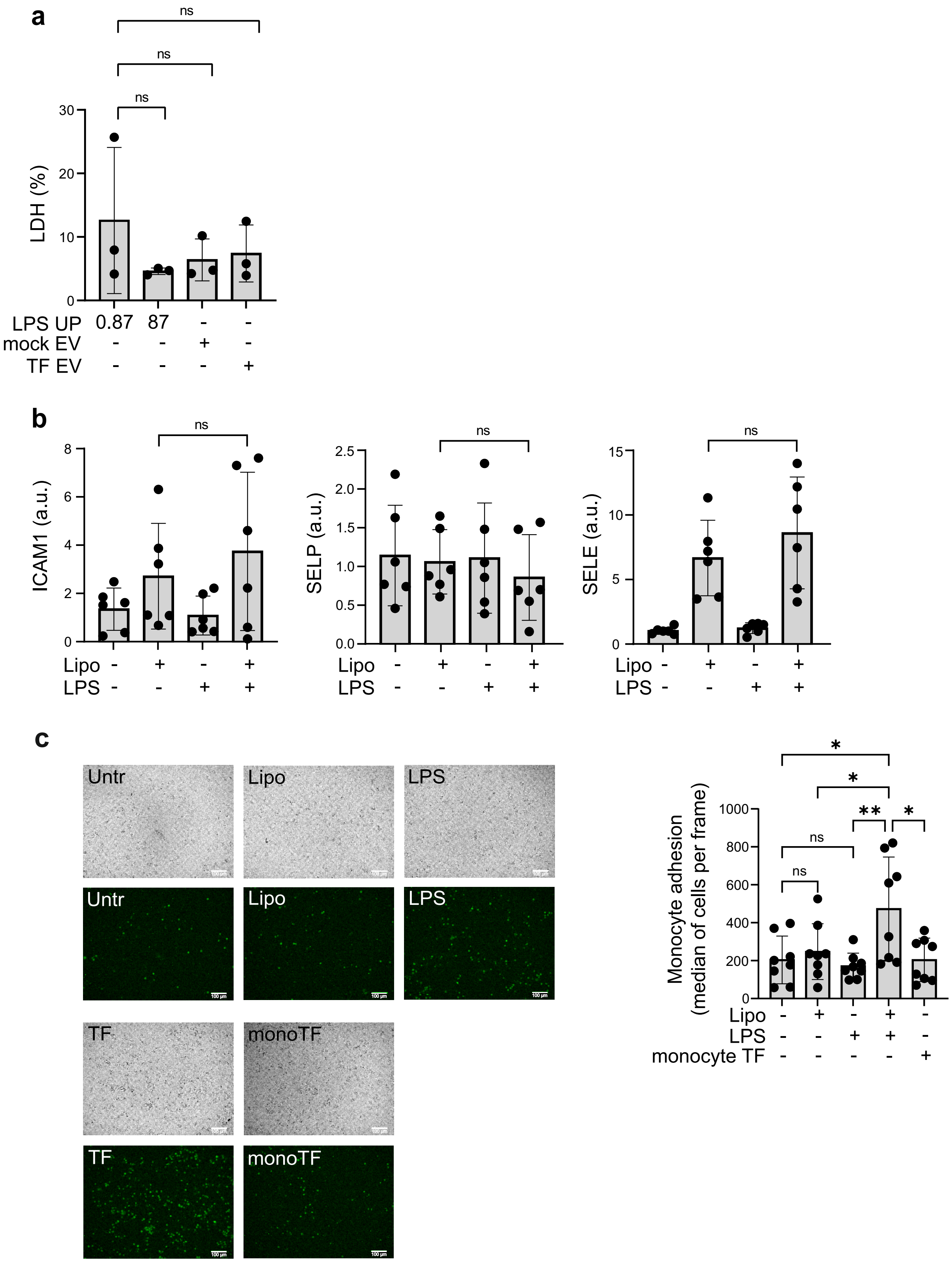

Fig. S6 Extracellular vesicles from activated endothelial cells do not induce cell death in human primary macrophages
a) Human primary macrophages were stimulated with HCAEC EVs from LPS or mock transfected HCAECs. As control human primary macrophages were stimulated with the same amount of ultrapure LPS (0.87 EU) that was detected on EV preparate and with 100x higher concentration. The secretion of LDH was detected by LDH assay. **b)** HCAECs were transfected with a complex of ultra-pure LPS (2 µg/ml) and lipofectamine (5 µl/ml), mock-treated with lipofectamine (5 µl/ml) or stimulated with extracellular ultra-pure LPS (2 µg/ml) for 6 h and expression of adhesion molecules *ICAM1*, *SELP*, and *SELE* was detected by RT qPCR. **c)** HCAECs were transfected with a complex of ultra-pure LPS (2 µg/ml) and lipofectamine (5 µl/ml), mock-treated with lipofectamine (5 µl/ml) or extracellular ultra-pure LPS for 2 h and fluorescently labelled (FITC) THP-1 monocytes were let to adhere for 1 h. As a control treatment (monocyte TF), transfection complexes were also added to THP-1 monocytes for the time of the adherence (1 h). Adherent monocytes were detected by fluorescent microscopy, quantifications and representative images shown. Statistics: a-c) One-way ANOVA followed by Sidak´s multiple comparisons test a) n=3, b) n=6, c) n=8.
